# Multivalency governs HP1α association dynamics with the silent chromatin state

**DOI:** 10.1038/ncomms8313

**Published:** 2015-06-18

**Authors:** Sinan Kilic, Andreas L. Bachmann, Louise C. Bryan, Beat Fierz

**Affiliations:** 1 Laboratory of Biophysical Chemistry of Macromolecules, Institute of Chemical Sciences and Engineering, Ecole Polytechnique Fédérale de Lausanne (EPFL), 1015 Lausanne, Switzerland

## Abstract

Multivalent interactions between effector proteins and histone post-translational modifications are an elementary mechanism of dynamic chromatin signalling. Here we elucidate the mechanism how heterochromatin protein 1α (HP1α), a multivalent effector, is efficiently recruited to the silent chromatin state (marked by trimethylated H3 at Lys9, H3K9me3) while remaining highly dynamic. Employing chemically defined nucleosome arrays together with single-molecule total internal reflection fluorescence microscopy (smTIRFM), we demonstrate that the HP1α residence time on chromatin depends on the density of H3K9me3, as dissociated factors can rapidly rebind at neighbouring sites. Moreover, by chemically controlling HP1α dimerization we find that effector multivalency prolongs chromatin retention and, importantly, accelerates the association rate. This effect results from increased avidity together with strengthened nonspecific chromatin interactions of dimeric HP1α. We propose that accelerated chromatin binding is a key feature of effector multivalency, allowing for fast and efficient competition for binding sites in the crowded nuclear compartment.

## Abstract (web summary)

Chromatin effector proteins often employ multivalent interactions with histone post-translational modifications. Here by using chemically defined nucleosome array and single-molecule microscopy, the authors show that effector multivalency prolongs chromatin retention and accelerates the association rate.

## Graphical abstract

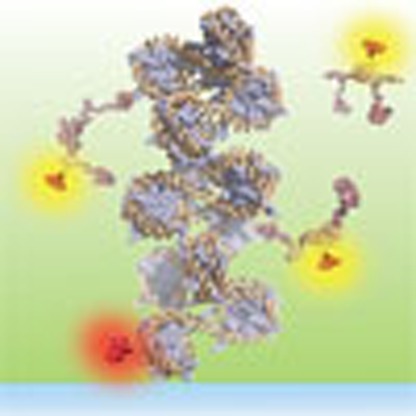

Chromatin effectors, carrying histone post-translational modification (PTM) binding domains (readers), are recruited to specific chromatin states where they alter chromatin structure, control gene expression or maintain genome integrity^1^,^2^. Dissociation constants for the interaction of individual reader domains with their cognate histone PTMs are relatively weak—often in the micromolar (μM) range^1^. Several models are conceivable to explain how chromatin effectors, despite their modest affinity, achieve efficient chromatin localization: First, the spatial co-existence of many binding sites in chromatin fibres allows rapid rebinding after dissociation. Second, oligomerization of effectors along the chromatin fibre can result in the formation of stable and long-lived complexes. Third, effectors often contain several binding domains or exist in homo- or hetero-oligomeric complexes. This allows simultaneous participation of several PTMs in multivalent binding interactions, resulting in strongly increased affinity. For most effectors it is, however, not known to which degree these putative mechanisms contribute to efficient recruitment to chromatin.

Here, we investigate the relevance of each of these proposed models for the mechanism of chromatin recruitment of HP1α, a multivalent effector. Proteins of the HP1 family are structural effectors that bind H3K9me3 (ref. 3) and are involved in chromatin compaction^4^, gene repression, regulation of viral latency^5^ and control of transposable elements^6^. Of the three human HP1 isoforms (α, β and γ), HP1α and β are associated with stably repressed heterochromatin at telomeres and centromeres, regions that are visible as dense chromatin foci in mammalian cells^7^. HP1 proteins bind to H3K9me2/3 via an N-terminal chromodomain (CD) with a dissociation constant (*K*_D_) of 1–10 μM, depending on the subtype and species^8^,^9^,^10^. Further, in the *S. pombe* HP1 homologue Swi6, the CD has been found to form higher-order oligomers on chromatin binding^11^,^12^. In addition to the CD, HP1 contains a chromoshadow domain (CSD) responsible for protein dimerization^13^,^14^ and interaction with other proteins through PxVxL, PxVxI or related motifs^15^,^16^. A flexible, positively charged hinge region connects the CD and CSD and binds non-specifically to DNA as well as to RNA^17^,^18^, and these interactions have been implicated in heterochromatin maintenance^17^,^19^. In the nucleus, heterochromatin foci are stable over cell generations but exhibit fast dynamics on the individual protein level with HP1 residence times in the milliseconds-to-seconds timescale^20^,^21^. Moreover, HP1 is quickly released from chromatin sites in response to H3S10 phosphorylation during mitosis^22^,^23^, as a reaction to DNA damage^24^, and during the repression of heterochromatic transcripts^18^. It is not well understood how HP1α is efficiently recruited to heterochromatin sites enacting stable gene silencing, while maintaining a highly dynamic complex.

To investigate the recruitment and exchange dynamics of HP1 to modified chromatin fibres, highly sensitive experimental methods are required. Recent progress has been achieved in engineering synthetic chromatin fibres^25^ and in developing single-molecule techniques to analyse molecular ensembles *in vitro*^26^. Here we establish a fully chemically defined assay system to directly observe the real-time interaction dynamics of individual HP1α molecules with their target histone PTMs within their native context, nucleosome arrays. This approach is highly complementary to measurements of chromatin effector dynamics in cells, as we have complete control over the chromatin architecture and modification status. The methodology allows us to monitor stochastic HP1α—chromatin interaction events in equilibrium. A high local H3K9me3 density results in a prolonged HP1α residence time due to rapid rebinding on dissociation. Employing a novel chemical strategy to control the dimeric state of HP1α, we discover that the increased avidity of a multivalent effector not only further prolongs its residence time, but also that it results in accelerated chromatin association. Importantly, we also observe this behaviour in the presence of PxVxI motif-containing interaction partners, which stabilize the HP1α dimeric state. High affinity of HP1α for H3K9me3-modified chromatin thus results from a fast chromatin association rate, while rapid local dissociation and rebinding kinetics ensure the maintenance of the dynamic complex required for heterochromatin function.

## Results

### HP1α dynamically binds H3K9me3 in chromatin

**Figure 1:**
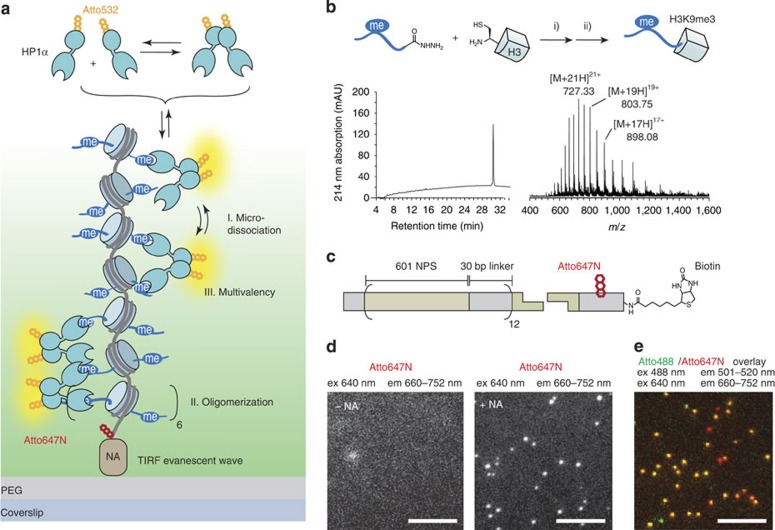
An assay to measure effector interaction dynamics with chromatin arrays. (**a**) Scheme of the experiment: Atto532-labelled HP1α dynamically interacts with immobilized H3K9me3-modified designer chromatin arrays, while the dynamic interactions are detected by smTIRFM. (**b**) Synthesis of H3K9me3 using a traceless EPL approach: (i) Oxidation of the hydrazide, *in situ* thioester formation and EPL reaction, (ii) desulfurization. Left: RP-HPLC analysis of the final protein, right: ESI-MS analysis of the final histone (MW: 15,251.8 Da calculated, 15,252 Da observed). (**c**) Design of chromatin DNA: recombinantly produced DNA carrying twelve 601-nucleosome position sequences (NPS) separated by 30 bp linkers, and a single, unique restriction site is ligated to a synthetic biotinylated and Atto647N-labelled oligonucleotide. (**d**) Chromatin array immobilization is specific and depends on neutravidin anchoring (−NA, neutravidin absent, +NA, neutravidin present) as observed by Atto647N fluorescence emission. Scale bars, 5 μm. (**e**) Atto488-labelled H2A, incorporated into the arrays, co-localizes with Atto647N-labelled DNA (orange spots).

We set out to investigate HP1α binding to H3K9me3 carrying chromatin fibres by smTIRFM, employing a chemically defined model system (Fig. 1a). First, we required access to chromatin fibres containing H3K9me3. We thus synthesized H3K9me3 by traceless expressed protein ligation (EPL) 27,28,29 (Fig. 1b and Supplementary Fig. 1) and reconstituted H3K9me3-containing or unmodified histone octamers using recombinant human histones (Supplementary Fig. 2). In parallel, we generated a DNA template for chromatin array reconstitution. To this end, recombinant DNA containing 12 copies of the 177 base-pair ‘601' nucleosome positioning sequence^30^,^31^ was ligated to a small oligonucleotide carrying a fluorescent dye (Atto647N) for localization, as well as biotin for immobilization on a solid support (Fig. 1c and Supplementary Fig. 3). Together with histone octamers containing either unmodified H3 or H3K9me3, chromatin fibres were reconstituted following established protocols^32^ (Supplementary Figs 2 and 3). These chromatin arrays were then specifically immobilized on poly-ethylene glycol (PEG)-passivated coverslips^33^ by biotin-neutravidin anchoring and imaged on a single-molecule level (Fig. 1d). To verify that the chromatin arrays remained intact on immobilization, we produced arrays with incorporated Atto488-labelled H2A. The Atto488 fluorescence emission co-localized with Atto647N-labelled DNA and no histone loss was detected over the time of a typical experiment (> 1 h) (Fig. 1e), indicating that the arrays were stable under single-molecule conditions.

**Figure 2:**
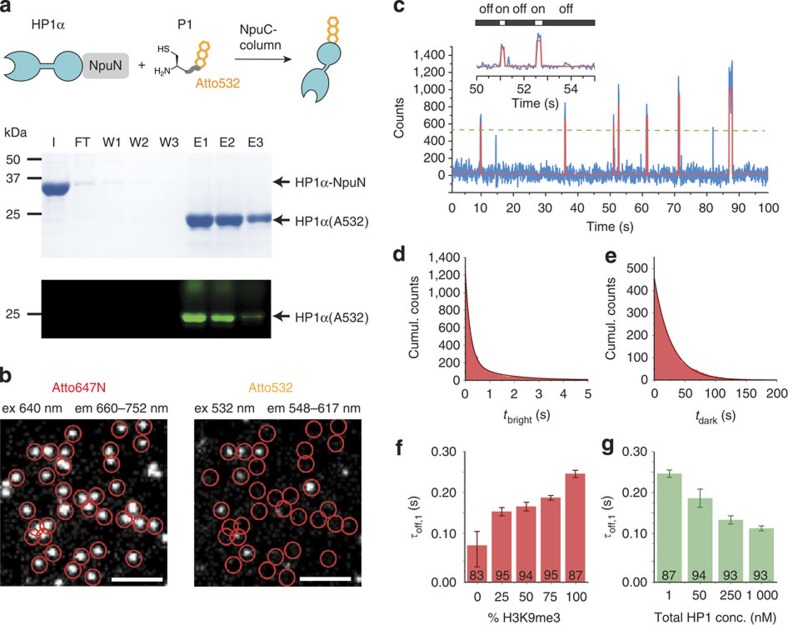
HP1α dynamically interacts with chromatin fibres. (**a**) HP1α fused to NpuN is expressed, bound to NpuC-beads, cleaved in ligation buffer and reacted with **P1** *in situ*, yielding labelled HP1α (HP1α(A532)). (I=input; FT=flowthrough; W1-3=washes; E1-3=elutions). (**b**) Left: single chromatin arrays are detected by Atto647N emission. Right: HP1α interaction dynamics are monitored by Atto532 emission. Scale bar, 5 μm. (**c**) Time trace of fluorescence intensity (blue) from a single chromatin array showing transient HP1α binding events, fitted by a step function (red). Inset: determination of bound (on-) and unbound (off-) times by a thresholding algorithm. (**d**) Dissociation kinetics: cumulative histogram of binding intervals (*t*_bright_) for 100 chromatin arrays (10^5^ frames each), fitted by a double-exponential function (fit: *τ*_off,1_=0.25±0.03 s, *τ*_off,2_=2.26±1.22 s). (**e**) Association kinetics: cumulative histogram of intervals between binding events (*t*_dark_) over 30 chromatin arrays, fitted by a single-exponential function (fit: *λ*_on_=22.9±9.8 s). (**f**) The HP1α residence time (*τ*_off,1_) depends on H3K9me3 density. Numbers indicate % amplitude of the fast phase (errors: s.e.m.; *n* =2–16 replicates). (**g**) The HP1α residence time (*τ*_off,1_) decreases as a function of HP1α concentration due to local competition. The indicated concentration of unlabelled HP1α is added to 1 nM Atto532-labelled HP1α. Numbers indicate % amplitude of the fast phase (errors: s.e.m., *n*=3–16 replicates). (**d–g**) For all fit results, see Table 1.

Second, to detect individual binding events we had to label HP1α with suitable fluorophores. To minimize structural perturbations of the protein, we decided to employ an EPL-based C-terminal labelling strategy. In a one-pot reaction, recombinant HP1α fused to the N-terminal part of the *N. punctiforme* (Npu) split-intein was converted into a C-terminal thioester^34^ followed by *in situ* ligation to a tripeptide carrying an Atto532 fluorophore (**P1**, Fig. 2a and Supplementary Fig. 4), resulting in a labelling efficiency of up to 75%. The dissociation constant (*K*_D_) of the labelled HP1α and a K9me3 containing H3 peptide was determined to be 5.36±1.33 μM, similar to unlabelled HP1α (Supplementary Fig. 5) and in agreement with literature values^4^.

Having all necessary components in hand, we then proceeded to measure HP1α—chromatin interaction dynamics using smTIRFM. To this end, 1 nM Atto532-labelled HP1α was injected into flow-cells containing immobilized, H3K9me3-modified chromatin arrays. Positions of single chromatin arrays were detected by Atto647N emission, whereas HP1α binding dynamics were monitored by Atto532 emission (Fig. 2b), revealing rapid transient chromatin interactions (Fig. 2c). A kinetic analysis of the resulting dwell-time histograms revealed double-exponential dissociation kinetics, with a fast major phase decaying with a time constant of *τ*_off,1_=0.25±0.03 s (±s.d.) and a slower process with *τ*_off,2_=2.30±0.88 s (Fig. 2d, for all measured kinetic constants, refer to Table 1). These time constants are comparable to HP1α dwell times observed in living cells^21^. Furthermore, the observed dwell times were not limited by fluorophore photobleaching, as HP1α-bound Atto532 decayed with a time constant >40 s under typical imaging conditions (Supplementary Fig. 6).

Conversely, analysing the time intervals between binding events yielded an apparent association time constant of *λ*_on_=22.9±9.8 s (Fig. 2e). From *λ*_on_ (taking into account the HP1α concentration and the number of nucleosomes per array) a microscopic association rate constant of *k*_on_=3.58 × 10^6^ M^−1^ s^−1^ was determined, resulting in an apparent *K*_D_=1.1 μM for a nucleosome in an array. This association rate constant is within the expected range of 10^5^–10^6^ M^−1^ s^−1^ for diffusion-controlled binding reactions of proteins^35^. We then repeated the experiment using unmodified (H3K9me0) chromatin arrays. In the absence of the mark, HP1α binding was almost completely abolished, and the few binding events decayed with fast kinetics (Fig. 2f and Supplementary Fig. 7). In agreement, a CD mutant of HP1α (W40A) exhibited very few binding events and fast off-rate constants, even in the presence of fully methylated arrays (Supplementary Fig. 7). These results indicate that we indeed directly measure histone PTM-dependent interactions while non-specific binding events are rare.

### HP1α undergoes rapid dissociation and rebinding

Having established a system that allows the quantitative determination of the HP1α interaction kinetics with modified chromatin arrays, we proceeded to test different models of interaction (see Fig. 1a). First, to assess the possibility that HP1α undergoes a series of dynamic microdissociation and rebinding events, we investigated the kinetic effect of reducing the number of available binding sites. We thus assembled arrays containing different fractions of trimethylated H3K9. The stochastic binding dynamics of HP1α to those arrays revealed that the residence time decreases with lower methylation density (Fig. 2f), indicating that rapid dissociation and rebinding events indeed contribute to the apparent residence time observed in densely modified chromatin. Moreover, we performed HP1α-binding experiments in the context of single nucleosomes, which also resulted in faster dissociation kinetics, due to the lack of neighbouring H3K9me3 sites (Supplementary Fig. 7).

### HP1α molecules compete for H3K9me3 sites

Second, we probed the ability of HP1α to oligomerize along the chromatin fibre interactions, which should result in a prolonged retention of individual HP1α proteins. Such a model for propagation of heterochromatin has been proposed for the yeast homologue Swi6 (refs 11, 12). Under our standard measurement conditions the chromatin fibres are only occupied by single HP1α monomers and dimers, thus rendering the formation of larger oligomers impossible. To increase HP1α occupancy while retaining the possibility to observe single molecules, we therefore successively increased the total protein concentration in the flow cell by the addition of unlabelled HP1α. Contrary to our expectation, this resulted in a concentration-dependent decrease in *τ*_off,1_, up to 3.5-fold at 1 μM total HP1α concentration (Fig. 2g). This indicates that HP1α does not predominantly form higher-oligomeric complexes, but rather that individual proteins compete for binding sites. This local competition then prevents rapid rebinding of dissociated HP1α molecules, resulting in an apparent decrease in residence time, in a process termed facilitated dissociation^26^,^36^. Such concentration-dependent dissociation kinetics were recently observed for several DNA-binding proteins^37^,^38^.

### Multivalent chromatin interactions accelerate HP1α binding

Third, we explored the influence of multivalent chromatin interactions on HP1α recruitment. *K*_D_ values of 0.5–5 μM have been reported for CSD homodimerization of different HP1 subtypes^14^. Therefore, under single-molecule conditions (at 1 nM concentration) a large fraction of HP1α dimers is dissociated into monomers. Indeed, an analysis of the fluorescence intensities of individual HP1α binding events indicated that 17% of all observations could be attributed to dimeric proteins (Supplementary Fig. 9). First, we investigated whether disruption of any residual dimerization alters association dynamics. We therefore expressed and labelled an HP1α protein with a mutated CSD (I163E), which can no longer form dimers^13^ (Supplementary Fig. 4). In this protein, the function of the CD was not impaired (*K*_D_=9.4±2.8 μM for an H3K9me3 peptide, Supplementary Fig. 5), and chromatin residence times were only slightly reduced as compared with wild-type HP1α (Supplementary Fig. 7). In contrast, we observed an increased apparent association time *λ*_on_ (>100 s), demonstrating the requirement for CSD interactions for efficient chromatin binding.

**Figure 3:**
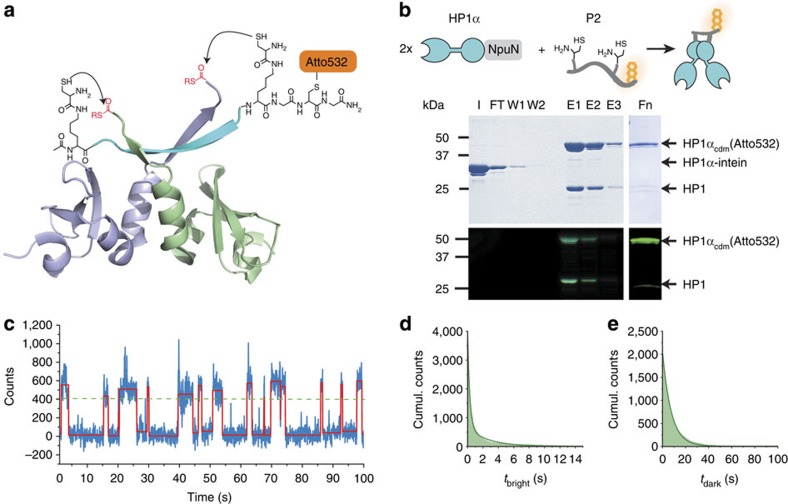
Affinity-directed HP1α dimerization allows investigating multivalent chromatin binding. (**a**) Strategy to synthesize HP1α_cdm_ employing the PxVxI peptide **P2** in an affinity-directed dual-EPL reaction (PDB: 3Q6S, ref. 39). (**b**) One-pot production of Atto532-labelled HP1α_cdm_: HP1α(1–177), fused to the NpuN-split intein was bound to NpuC-beads and eluted with ligation buffer containing the hSgoL1 peptide (carrying two cysteinyl-lysines and an Atto532 dye), yielding labelled HP1α_cdm_ (Atto532). Pure HP1α_cmd_ was obtained after gel-filtration purification. Gel annotation: I=input; FT=flowthrough of column; W1-2=column washes; E1-3=elution fractions; Fn=final protein after gel filtration. (**c**) Time trace of fluorescence intensity (blue) from a single chromatin array fitted by a step function (red) and showing transient HP1α_cdm_ binding events (at 0.5 nM). (**d**) HP1α_cdm_ dissociation kinetics (100 chromatin arrays, 10^5^ frames each) fitted by a double-exponential function (fit: *τ*_off,1_=0.33±0.01 s, *τ*_off,2_=3.40±0.53 s). (**e**) HP1α_cdm_ association kinetics over 30 chromatin arrays, fitted by a single-exponential function (fit: *λ*_on_=7.45±1.87 s). (**d**,**e**) For all fit results, see Table 1.

Second, to constrain HP1α in a dimeric state even under a low concentration regime, we developed a novel chemical strategy to produce covalently dimerized protein. In an affinity-directed dual-protein ligation reaction, two HP1α protomers with C-terminal thioesters, which were optimally placed at the CSD, were ligated to a PxVxI-containing peptide with high CSD affinity and two ligation handles (Fig. 3a). The HP1-binding sequence of the shugoshin protein (hSgoL1, residues 448–457)^39^ was chosen as a suitable sequence. hSgoL1 plays a role in the protection of centromeric sister-chromatids and interacts with HP1, both in cell division and interphase with different functions^39^,^40^. We thus synthesized a peptide (**P2**), based on the hSgoL1 PxVxI-motif, flanked by two lysines with a cysteinyl-residue attached to their side chains, and containing the Atto532-fluorescent dye for detection (Fig. 3a). On the HP1α CSD, installation of a C-terminal thioester at position 177 was assessed as being optimal for reactivity, and HP1α(1–177)-SR was prepared using the Npu split-intein. Subsequent *in situ* dual-ligation with **P2** resulted in covalently linked dimers (HP1α_cdm_, 60% yield), which were further purified to homogeneity by size-exclusion chromatography (Fig. 3b and Supplementary Fig. 8). Finally, peptide binding assays revealed that the purified HP1α_cdm_ is functional and binds to an H3K9me3 peptide with an affinity similar to HP1α (*K*_D_=12.5±0.5 μM, Supplementary Fig. 5).

We then proceeded to probe chromatin binding with HP1α_cdm_. At a concentration of 0.5 nM HP1α_cdm_ (equivalent to 1 nM HP1α monomers), we observed binding events of longer average duration and of increased frequency compared with HP1α (Fig. 3c). Analysing the intervals of bound and unbound HP1α_cdm_ revealed double-exponential dissociation kinetics (Fig. 3d), with *τ*_off,1_=0.33±0.01 s (87% amplitude) and *τ*_off,2_=3.40±0.53 s (13% amplitude), and single-exponential binding kinetics with *λ*_on_=7.45±1.87 s (Fig. 3e).

**Figure 4:**
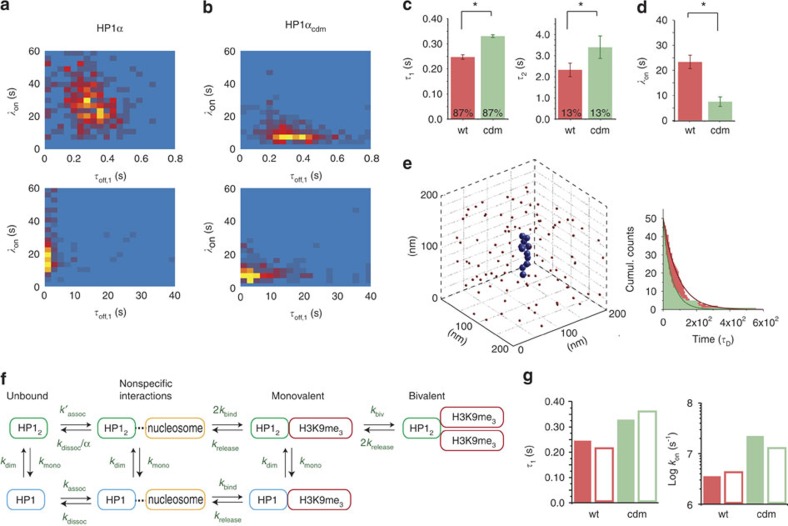
Multivalent HP1α-chromatin interactions increase association speed. (**a**) Distribution of interaction kinetics from HP1α with single chromatin arrays: Two dimensional histograms of single-array binding kinetics showing the correlation of dissociation time constants, *τ*_off,1_ and *τ*_off,2_ with the apparent association time constant *λ*_on_. (**b**) Two dimensional histogram of single-array binding kinetics for HP1α_cdm_. (**c**) Comparison of the average dissociation time constants *τ*_off,1_ and *τ*_off,2_ (fast and slow phase) between HP1α and HP1α_cdm_ (errors: s.e.m., *n*=4–16 replicates, *P<0.05, Student's *t*-test). (**d**) Comparison of the association rate constant *k*_*on*_ between HP1α and HP1α_cdm_ (errors: s.e.m., *n*=4–16 replicates, *P<0.05, Student's *t*-test). (**c**,**d**) For all fit results, see Table 1. (**e**) Brownian dynamics simulation of chromatin binding: monomeric or dimeric binders are diffusing in a box with 200 nm edge length containing a 12-nucleosome chromatin array. First passage binding kinetics are plotted for monomeric (red) and dimeric (green) binders at 20 and 10 μM binder concentration, respectively. Single-exponential fits yield *λ*_on_=53.6 *τ*_D_ for the dimeric and *λ*_on_=89 *τ*_D_ for the monomeric binder (*τ*_D_ is the characteristic diffusion time, *τ*_D_*=6πηa*^*3*^*/(k*_B_*T)* for binders with radius *a* in solvent with viscosity *η, k*_B_*T* being the thermal energy). (**f**) Kinetic model for chromatin binding of HP1α: HP1α in a monomer-dimer equilibrium (*k*_mono_, *k*_dim_) associates nonspecifically (*k*_assoc_) and reversibly (*k*_dissoc_) with nucleosomes (short-lived interactions with (i) the nucleosomal or linker DNA, (ii) the nucleosomal surface or (iii) different regions on histone tails) before transitioning to a specific, H3K9me3 bound state (*k*_bind_) with a 100 ms-residence time (*k*_release_). The nonspecific association of dimeric protein is prolonged by a factor α, and it can further proceed to a bivalent state (*k*_biv_). (**g**) Comparison of experimental data of HP1α and HP1α_cdm_ chromatin binding with results from kinetic model in (**f**). For parameters, see Supplementary Table 1.

To analyse the effect of HP1α dimerization on a single-array level, we generated two-dimensional histograms of *τ*_off,1_ and *τ*_off,2_ versus *λ*_on_, where each data point originated from the analysis of the kinetic trace originating from an individual chromatin array. Inspecting the histograms for HP1α (Fig. 4a) and HP1α_cdm_ (Fig. 4b) we observed a shift of the distribution to longer residence times and shorter *λ*_on_ for the dimeric protein, as well as significant variation between the individual chromatin fibres. This variation may arise from local heterogeneity in nucleosome array structure, including dynamic conformational transitions, influencing the HP1 interaction modes.

From these measurements, it can be concluded that HP1α dimerization directly results in a significant increase in chromatin residence time (Fig. 4c). This effect can be attributed to the increased possibility of simultaneous engagement of two K9me3-bearing H3-tails in the chromatin array, the inability of dimers dissociating into monomers, as well as an increased chance of rebinding upon transient dissociation for HP1α_cdm_. Importantly, the dimeric HP1α_cdm_ also exhibited significantly more rapid chromatin binding, with *k*_on_=(2.24±0.60) × 10^7^ M^−1^ s^−1^ (Fig. 4d). This corresponds to a sixfold increase in the association rate constant for HP1α_cdm_ compared with HP1α, and results in an apparent *K*_D_ of 0.14 μM, about 60-fold increased affinity compared with isolated H3 peptides (Supplementary Fig. 5).

To gain further insight into the molecular mechanism of how multivalent binders can speed up chromatin recruitment, we performed computational modelling. To explore the kinetic processes governing individual binding events, we implemented a coarse-grained computational model^36^ (Supplementary Fig. 10 and Supplementary Methods). Simulated association kinetics from a Brownian dynamics simulation of monomeric or dimeric binders interacting with nucleosomal arrays could reproduce an increase in association rate constant for the dimeric factors, compared with monomers (fourfold, Fig. 4e). An analysis of the trajectories revealed that the increase in association speed for multivalent binders arises mainly from presenting multiple interaction domains, in combination with enhanced non-specific chromatin interactions in the dimers. These nonspecific interactions increase the time dimeric binders spend in the vicinity of chromatin, thereby raising the probability of a productive encounter.

These findings then allowed us to formulate a kinetic model to describe our experimental data (Fig. 4f). Stochastic simulations based on this kinetic model reproduced the measured major kinetic phases well (Fig. 4g, for parameters, see Supplementary Table 1). In agreement with the Brownian dynamics simulation, a rapid pre-equilibrium of nonspecific HP1α—chromatin interactions results in increased association kinetics for the dimeric protein if the release rate constant *k*_dissoc_ is reduced for the dimer (α=5). In addition, the obtained microscopic rate constants for bivalent binding revealed that, at a given time, only a small fraction of dimeric HP1α simultaneously engages two H3K9me3 marks.

### Induced HP1 dimerization drives chromatin association

PxVxI/L containing peptides have been shown to induce HP1α dimerization^41^. Correspondingly, proteins interacting with the CSD have been implicated in stabilizing HP1–chromatin interactions^16^,^42^.

**Figure 5:**
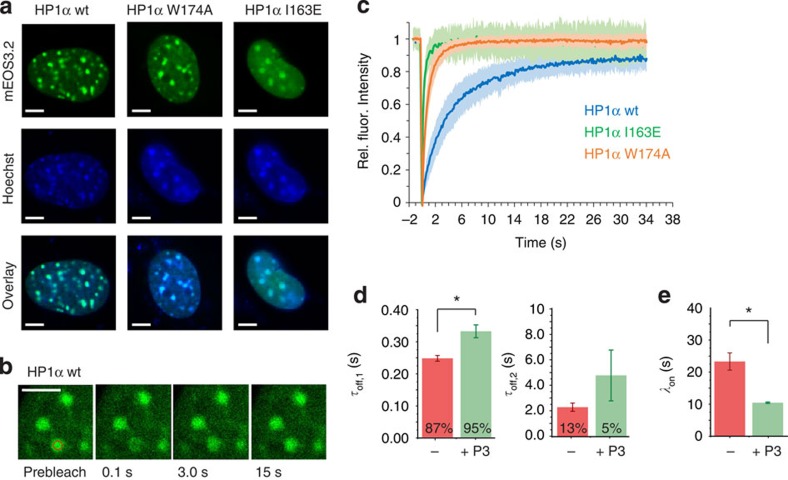
CSD interactions accelerate HP1 association dynamics. (**a**) Confocal flurescence images of NIH 3T3 cells transfected with mEos3.2-HP1a, mEos3.2-HP1α(I163E) or mEos3.2-HP1α(W174A) and overlay with Hoechst staining. Scale bar, 5 μm. (**b**) Time series of images from a typical FRAP experiment (HP1α), showing the bleached spot as a red circle pre- and post bleach at indicated times. Scale bar, 5 μm. (**c**) FRAP analysis of mEos3.2-HP1α wt or W174A in heterochromatin foci (*n*=15 cells with two foci from each, the shaded area denotes the standard deviation at each time point). Analysis of the traces using a diffusion/binding model^46^ results in the following values: HP1α: *λ*_on_=1.2±0.4 s and *τ*_off_=2.8±1.2 s, HP1α(W174A): *λ*_on_=1.0±0.2 s and *τ*_off_=1.1±0.3 s, HP1α(I163E): *λ*_on_=1.8±0.3 s and *τ*_off_=0.8±0.1 s. (**c**) Comparison of the average dissociation time constants *τ*_off,1_ and *τ*_off,2_ (fast and slow phase) between HP1α in the absence and presence of **P3** (error bars: s.e.m., *n*=4–16 replicates, *P<0.05, Student's *t*-test). (**d**) Comparison of the average dissociation time constants *τ*_off,1_ and *τ*_off,2_ (fast and slow phase) between HP1α in the absence and presence of **P3** (error bars: s.e.m., *n*=4–16 replicates, *P<0.05, Student's *t*-test). (**e**) Comparison of the association rate constant *k*_on_ between HP1α in the absence and presence of **P3** (errors: s.e.m., *n*=4–16 replicates, *P<0.05, Student's *t*-test). (**c,d**) For all fit results, see Table 1.

Thus, we investigated whether interactions at the CSD influence the chromatin association dynamics of HP1α in cells. To this end we measured fluorescence recovery after photobleaching (FRAP) curves for wild-type and two mutant HP1α variants (I163E and W174A) fused to the monomeric fluorescent protein mEos3.2 (ref. 43). While the I163E mutant protein is strictly monomeric, the W174A mutation disrupts the interaction between the CSD and PxVxI/L ligands^13^ and therefore abolishes ligand-induced dimerization. Transfected into NIH 3T3 fibroblasts, all proteins produced discernable heterochromatin foci, which matched to regions of dense chromatin as observed by DNA staining (Fig. 5a). FRAP measurements within heterochromatin foci revealed that the complete loss in dimerization with the I163E mutant resulted in highly impaired chromatin binding, whereas also the W174A mutant exhibited reduced affinity, faster dynamics and a larger mobile fraction compared with wild-type HP1α (Fig. 5b,c). This reveals the importance of CSD interactions, which favour HP1α dimerization as well as organize HP1α into larger complexes, and thus are important for efficient chromatin recruitment and stable association.

On the basis of these results, we tested whether we could reproduce the effect of CSD binding proteins on HP1α chromatin association kinetics in our *in vitro* system. We thus synthesized a further variant of the PxVxI sequence of hSgoL1 (448–457) (**P3,** which binds HP1α with a *K*_D_=110 nM, Supplementary Fig. 11), and measured HP1–chromatin interactions in the absence and presence of 1 μM **P3**. The resulting interaction dynamics showed both an increased residence time and significantly accelerated association when **P3** was present (Fig. 5d,e). In agreement, the fluorescence intensity distribution of individual observations in the smTIRF traces showed a 2.5-fold increase in HP1α dimerization (42% dimeric HP1α) upon peptide addition (Supplementary Fig. 9). Importantly, a diminished effect on chromatin association kinetics was measured when a mutated peptide, **P4**, was added (same sequence as **P3** with the PxVxI motif mutated to A, Supplementary Fig. 11, due to its inability to induce HP1α dimerization. A remaining nonspecific effect on the observed kinetics most likely originates from direct DNA interaction of the peptides, due to their poly-cationic character. Thus, protein–protein interactions at the CSD, which induce HP1α dimerization and thereby multivalency, result in prolonged retention and accelerated chromatin association.

## Discussion

Histone PTMs are read out by effector proteins or protein complexes, which often contain multiple reader domains. The increased avidity of these complexes allows multivalent engagement of multiple spatially confined marks in a chromatin fibre, thereby not only increasing the affinity towards a certain chromatin state but also providing a mechanism for combinatorial readout of co-existing histone PTMs^44^. Even when forming stable structures such as heterochromatin, chromatin effectors like HP1α exist in a dynamic equilibrium between chromatin-bound states and an unbound pool of proteins^20^, thereby allowing a rapid response to external stimuli. However, how these effectors can be efficiently and stably recruited to a particular chromatin state while retaining fast exchange dynamics has remained an unsolved question. In living cells, FRAP and related photobleaching methods, as well as fluorescence correlation spectroscopy-based approaches have resulted in significant insight into the kinetic chromatin association processes for a large number of effectors^45^. However, a direct mechanistic interpretation is complicated by the fact that the chromatin state (amount, density and combinations of histone PTMs) at the observed nuclear binding sites is neither known nor can it be controlled in an experiment. Furthermore, the determination of kinetic parameters from FRAP or fluorescence correlation spectroscopy requires the application of mathematical models^46^,^47^, which can lead to misinterpretations of the kinetics if not done carefully.

Here, we thus developed a general and complementary method, combining direct single-molecule detection with chemically defined chromatin fibres and purified effectors. This allowed us to directly observe interaction dynamics of single HP1α molecules with H3K9 methylated chromatin arrays of controlled architecture and modification status. Furthermore, by employing chromatin arrays as binding substrate we could explore effects arising from the local accumulation of histone PTMs in their native chromatin environment. Our measurements of HP1α dynamics revealed double-exponential dissociation kinetics whose major kinetic phase exhibited a residence time in the 100–300 ms range. The detected residence times were shown to depend on the density of H3K9me3 on the chromatin arrays. This indicates that dissociation is not a monomolecular process but includes multiple microdissociation and rebinding events before final release. In addition, a slower process on the seconds timescale was detected, which may arise from multivalent interactions. In fact, HP1α dynamics in living cells show a similar complex behaviour with multiscale kinetics^21^. Of note, in the cell, chromatin arrays are less homogenous compared with the model arrays used in the current study. Future studies are thus required to dissect how variability in the chromatin architecture (including variability in DNA sequence, linker DNA length or nucleosome occupancy) and internal chromatin dynamics influences effector binding kinetics.

Interestingly, raising the HP1α concentration reduced the residence time of bound molecules in spite of increased chromatin occupancy. These findings argue against the presence of additional interactions between HP1α molecules besides CSD-mediated dimerization, and thus the formation of higher-order oligomers along the chromatin fibre is unlikely. Indeed, yeast Swi6, for which such a mechanism was proposed^11^,^12^, contains significant sequence differences compared with human HP1α, in particular in an extended α-helix in its CD as well as in a lysine-containing loop, which both were proposed to be an additional site of interaction. Rather, the data point towards a mechanism involving facilitated dissociation, resulting from competition for available binding sites, a common feature also recently described for DNA interacting proteins^26^,^36^,^37^.

Simultaneous interactions of dimeric HP1α with two H3 tails have been proposed to induce chromatin compaction^4^, and such multivalency effects are expected to result in stable chromatin association of effectors in general^48^. To control the oligomeric state of HP1α at the low concentration required for single-molecule measurements, and thus to put these models to the test, we devised a new affinity-directed dual-ligation approach to covalently dimerize HP1α. Indeed, these molecules exhibited significantly faster association kinetics and longer residence times. Importantly, an increase in association kinetics could also be induced by the addition of a CSD-binding PxVxI-containing peptide derived from the HP1α-interacting protein hSgoL1. In agreement, CSD interacting proteins have been known to increase the equilibrium binding affinity of HP1α^42^.

Taken together, our results suggest that the increased affinity upon HP1α dimerization arises mainly through strengthening of the association rate constant. A strong increase in binding rate, as opposed to an effect on dissociation kinetics, allows to increase the local concentration of HP1α at chromatin, while the complex remains very dynamic. In addition, in a crowded environment such as chromatin with high levels of competition for binding sites, fast binding kinetics are of critical importance^35^. Kinetic modelling provided a molecular explanation of the accelerated binding kinetics as a result from a strengthened non-specific encounter complex and increased probability of H3K9me3 binding for the dimeric molecule due to the presentation of two CDs. In contrast, we can exclude a direct allosteric activation of the CD through CSD dimerization, as both the monomeric HP1α(I163E) and the covalent dimeric HP1α_cdm_ exhibit comparable H3K9me3 peptide affinities compared with HP1α in equilibrium titration experiments.

In the cell, HP1α exists at a concentration of around 0.5–1 μM (ref. 21), depending on the chromatin compartment, which is close to the *K*_D_ of the CSD domains (1.5–3 μM)^14^,^49^. Solely, on the basis of this value a significant monomeric fraction of HP1α is expected. This fraction is, however, efficiently converted into dimeric and multivalent effectors upon interaction with binding partners associating in the CSD binding cleft. In agreement with this, our *in vivo* FRAP data show that HP1α mutants carrying CSDs unable to interact with protein ligands are no longer able to stably associate with chromatin, presumably because they are unable to efficiently compete with wild-type HP1 molecules. Thus, the recruitment of HP1-interacting proteins represents an added layer of heterochromatin regulation through controlling multivalency and kinetics of HP1α binding and exchange. Finally, HP1α proteins are subject to PTMs including phosphorylation, acetylation^50^ as well as SUMOylation^51^, which might alter protein–protein and protein–DNA interactions and further fine-tune the dynamic recruitment of HP1α. We expect that, in the competitive chromatin environment, effector binding kinetics critically determine the readout of single and combinatorial histone PTMs, and thus that effector multivalency may be a general strategy to control dynamic protein recruitment to chromatin.

## Methods

### EPL to generate H3 K9me3

Synthesis of H3 1–14 (K9me3) peptide hydrazide was performed on a pre-formed hydrazide resin using a standard fluorenylmethoxycarbonyl (FMOC) Nα protection strategy on an automated peptide synthesizer. In a typical ligation, 3 μmol of H3 1–14 (K9me3) peptide hydrazide (5.0 mg) was dissolved in ligation buffer (6 M GdmCl, 200 mM phosphate, pH 3.0 at RT) to a final concentration of 5 mM and the pH readjusted to 3.0. The peptide solution was kept at −10 °C in an ice/KCl bath and NaNO_2_ was added dropwise from a 200 mM stock solution to a final concentration of 15 mM. After mixing, the peptide was left at −10 °C for 20 min. A total of 0.75 μmol of the protein was dissolved in mercaptophenyl acetic acid (MPAA) ligation buffer (6 M GdmCl, 200 mM phosphate, 300 mM MPAA, pH 8.0) to a final concentration of 1.2 mM. After 20 min of *in situ* activation the peptide was brought to room temperature and the solution of protein in MPAA ligation buffer mixed with the activated peptide. The pH was readjusted to 7.7–7.9 and ligation allowed to proceed until completion as determined by RP-HPLC (Supplementary Fig. 1c). When the reaction had reached completion, TCEP was added, the reaction was mixed with two volumes of RP-HPLC solvent 20% B and purified by semipreparative RP-HPLC using a gradient of 0–70% B over 45 min. The ligation product was analysed by RP-HPLC and ESI-MS (Calculated MW=15,283.9 Da, observed MW=15,285.0 Da) (Supplementary Fig. 1d).

### Desulfurization to convert H3K9me3 A15C to H3K9me3

The ligation product was dissolved in TCEP desulfurization buffer (6 M GdmCl, 200 mM phosphate, 250 mM TCEP, pH 6.5) to a final concentration of 0.5 mM. Glutathione in TCEP desulfurization buffer and VA-044 radical starter were added from stock solutions to the ligation product to give final concentrations of 40 and 20 mM, respectively. The pH was readjusted to 6.5 and the desulfurization allowed to proceed at 37 °C. The reaction was continuously monitored by RP-HPLC and ESI-MS. When the reaction was complete, two volumes of RP-HPLC solvent (20% B) were added and the product purified by semipreparative RP-HPLC using a gradient of 0–70% B over 45 min. The protein was finally analysed by RP-HPLC and ESI-MS (Fig. 1b) (Calculated MW=15,251.8 Da, observed MW=15,251.0 Da).

### Histone octamer refolding

In a typical octamer refolding reaction, 0.4 mg of each of the pure lyophilized human histones were dissolved in unfolding buffer (6 M GdmCl, 10 mM Tris, 5 mM DTT, pH 7.5). The exact concentration was determined by UV spectroscopy, using the following extinction coefficients: *ɛ*_280nm,H2A_=4,470 M^−1^ cm^−1^, *ɛ*_280nm,H2B_=7,450 M^−1^ cm^−1^, *ɛ*_280nm,H3_=4,470 M^−1^ cm^−1^, *ɛ*_280nm,H4_=5,960 M^−1^ cm^−1^. Equimolar amounts of H3 and H4 were then mixed along with 1.05 equivalents of H2A and H2B at 1 mg ml^−1^ and octamers were refolded by dialysis against refolding buffer (2 M NaCl, 10 mM Tris, 1 mM EDTA, 5 mM DTT, pH 7.5). The refolded octamers were subsequently purified by gel filtration on a Superdex S200 10/300GL column (Supplementary Fig. 2f,g). Collected fractions were analysed by sodium dodecyl sufate–PAGE (SDS-PAGE), and octamer containing fractions were pooled and concentrated to ∼50 μM octamer concentration. Finally, glycerol was added to a final concentration of 50%, concentrations were determined by UV spectroscopy and octamer stocks were stored at −20 °C.

### Generation of labelled and biotinylated chromatin array DNA

A plasmid containing a 12 × repeat of 601 nucleosome positioning sequence separated by 30-bp linker DNA segments (containing ScaI restriction sites) and a unique BsaI restriction site at the 3′ end was generated recombinantly, released from the vector backbone by EcorV digestion and purified by size-exclusion chromatography^31^. For labelling, 5 nmol of a synthetic oligonucleotide (5′-ph-CAGCTAGTCTGCT-3′ (amine-linker) 5′-CAGATATCGTCG-3′-Biotin), containing an internal amine attached to a dT and a C-terminal biotin, was mixed with 3 mM A647N-NHS ester (from a 30 mM stock in DMSO) in 0.1 M sodium tetraborate and incubated overnight at 4 °C. Subsequently, the labelled fragment was ethanol precipitated and the pellet was dissolved in 10 μl of deionized miliQ water and quantified. Then, the labelled oligonucleotide was annealed to its complementary DNA strand (5′-CGACGATATCTGAGCAGACTA-3′) in T4 DNA ligase reaction buffer (NEB). Subsequently, the labelled dsDNA oligonucleotide (5 eq. excess) was ligated to 12 × 601 array DNA using T4 DNA ligase for 1 h at room temperature (Supplementary Fig. 3a). The excess oligonucleotide was removed by purification with QIAquick spin columns (Qiagen), followed by PEG precipitation with 9% PEG 6000 (Supplementary Fig. 3a). Mononucleosome DNA was prepared using the same methodology, but employing a 1 × 601 NPS sequence. Concentrations and fluorescence were analysed by absorption spectrometry and fluorometry (Supplementary Fig. 3a).

### Chromatin and nucleosome reconstitution

Nucleosomes and chromatin arrays were reconstituted on a scale from 10 to 100 pmol (per mononucleosome) in 25–30 μl. 601 NPS DNA (1 or 12 repeats) was reconstituted in TE buffer (10 mM Tris, 0.1 mM EDTA, pH 7.5) and 2 M NaCl, followed by the addition of 1.1 equivalents of the respective histone octamers. For chromatin arrays, 0.5 equivalents of MMTV buffer DNA was included^31^. The reactions were dialysed gradually from TEK2000 buffer (TE buffer including 2000, mM KCl) to TEK10 buffer over 16 h, using a two-channel peristaltic pump. After dialysis, chromatin concentrations were determined by UV quantification. 0.5–2 pmol of chromatin arrays were digested with ScaI-HF (NEB) in 10 μl for 6 h. Quality of the reconstitution was assessed by native PAGE (Supplementary Fig. 3b,c). Chromatin and nucleosome reconstitutions were stored on ice for maximum 1 week after preparation.

### HP1α expression and labelling

Human HP1α was expressed in *E. coli* as a C-terminal fusion to the N-terminal half of the Npu split intein, followed by a hexahistidine tag. After purification by Ni-affinity and anion-exchange chromatography (to remove residual DNA), HP1α-NpuN was bound to a column of resin-linked NpuC. A Thz_1_-G_2_-C_3_-CONH_2_ tripeptide (**P1**) was synthesized by FMOC solid-phase peptide synthesis (Thz stands for thiazolidine). The peptide was subsequently reacted with Atto532-iodoacetamide in 20 mM Tris buffer, pH 7.5, followed by opening of Thz by incubation with 500 mM methoxylamine at pH 5. 1 mM purified peptide was then added to the column-bound HP1α in *in situ* EPL buffer (50 mM MPAA, 200 mM MESNA, 100 mM phosphate, 10 mM TCEP, 1 mM EDTA, pH 7.8). After overnight incubation at RT, the ligated protein was eluted, purified by size-exclusion chromatography, mixed with glycerol to 30% and flash frozen in small aliquots for later use.

### HP1_cdm_ synthesis

HP1α(1–177)-NpuN was expressed in *E. coli* and purified as described for HP1α-NpuN. The peptide **P2,** ac-K_1_S_2_L_3_Y_4_P_5_V_6_V_7_K_8_I_9_R_10_R_11_K_12_G_13_C_14_G_15_-CONH_2_, was synthesized, using alloc-protecting groups for lysines 1 and 12. Following specific deprotection of the alloc group, N-Boc-Thz was coupled to the lysines, followed by peptide cleavage, purification, Atto532-labelling using the iodoacetamide and final Thz opening by methoxylamine. The peptide was then added to intein-column-bound HP1α(1–177)-NpuN and reacted overnight at RT. The covalently dimerized HP1 was eluted and further purified by size-exclusion chromatography, mixed with glycerol to 30% and flash frozen in small aliquots for later use.

### Microscale thermophoresis titrations

Measurements were carried out on a MonoLith NT.115 equipped with blue/green filters. H3K9me3 1–14 peptide from a main stock was serially diluted in titration buffer (150 mM NaCl, 50 mM HEPES, 0.05% Tween, 1 mM DTT, pH=7.5) to cover the concentration range 0.1–100 μM and result in 10 μl samples of each. From a 300 nM stock (based on Atto532 absorption) of HP1α(A532) or HP1α_cdm_(A532), 10 μl was added to the serially diluted peptide. Samples were loaded into hydrophilic-coated capillaries and cap scanning done at 100% LED power. Thermophoresis was done at 80% IR laser power. Data were normalized between the start and the end point and fitted with a quadratic binding equation using Origin.

### Intrinsic tryptophan fluorescence titrations

A sample of 300 nM unmodified HP1α was loaded as 80 μl into an Ultra-micro fluorescence cuvette. Increasing concentrations of H3K9me3 1–14 peptide to cover the range 0.2–100 μM was titrated in as 1.5–7 μl per addition. Single-point measurements of the intrinsic tryptophan fluorescence were done for each titration point using 280 nm as excitation wavelength and 3 nm as the excitation slit width. Emission was measured at 348 nm with a slit width of 3.0 nm and an integration time of 0.5 s. The typical number of counts obtained was 50–70 k. The number of trials allowed per point was 5 or until a maximum s.d. of 0.5% was reached. The fluorescence counts measured were corrected for the dilution upon titration of the peptide and then normalized between the start and the end point. The normalized data were fitted with a quadratic binding equation using Origin.

### smTIRFM experiments

Glass coverslips and microscopy slides containing drilled holes were cleaned by sonication in 10% alconox, acetone and ethanol, followed by treatment with a mixture of concentrated sulfuric acid to 30% hydrogen peroxide (3:1). The water-rinsed and dried coverslips and slides were then silanized using 2% (3-aminopropyl)triethoxysilane in acetone and assembled into flow cells, containing four channels each separated by double-sided adhesive tape. Pipette tips were inserted into the drilled holes and the channels were sealed with epoxy glue. A solution of 100 mg ml^−1^ mPEG(5000)-succinimidyl carbonate containg 1% biotin-mPEG-succinimidyl carbonate was infused into the channels and reacted for 3 h, resulting in efficient passivation of all exposed surfaces in the channels. The channels were extensively washed using water and buffer T50 (10 mM Tris, 50 mM KCl) buffer before proceeding to experiments. For chromatin immobilization, 0.2 mg ml^−1^ neutravidin solution was infused using a high-precision syringe pump and incubated for 5 min, followed by extensive washes with T50. Then, 500 pM chromatin arrays in T50 buffer were injected into the neutravidin treated flow chamber for 5 min, followed by a wash with T50 and imaging buffer (50 mM HEPES, 130 mM KCl, 10% glycerol, 2 mM trolox, 0.005% tween-20, 3.2% glucose, glucose oxidase/catalase enzymatic oxygen removal system). Chromatin coverage was observed in the TIRF microscope (Nikon Ti-E) by fluorescent emission in the far-red channel upon excitation by a 640-nm laser line. Dynamic experiments were initiated by infusion of 1 nM Atto532-labelled HP1α or 0.5 nM HP1α_cdm_ in imaging buffer. All proteins were freshly diluted from a 100 nM stock and immediately injected to avoid changes in concentration due to adsorption to tube walls. HP1α dynamics were observed in the yellow/orange channel using a 530 nm laser line for excitation at 20 W/cm^2^ using an EMCCD camera (Andor iXon) at 20 frames/s for a 25 × 50 μm area at a resolution of 160 nm/pixels. Every 200 frames, an image of the chromatin positions in the far-red channel was recorded for drift correction.

### Data analysis

Movies of HP1α-chromatin interactions were recorded and converted into individual traces using a custom-made semi-automated Matlab (Mathworks) script: in short, after a global baseline-correction and drift-correction (using the far-red images interspersed at 200 frame intervals), individual chromatin array positions were determined using a peak-finding algorithm from the far-red images. Fluorescence intensity traces for each chromatin position were obtained by integrating over a circle of 2 pixel radius. Individual HP1 fluorescence peaks were included based on point-spread-function (PSF) and distance cutoffs. Kinetics were extracted from fluorescence traces using a semi-automated thresholding algorithm. Cumulative histograms were constructed from dark and bright intervals and fitted to sums of exponential functions.

### Cell culture

NIH/3T3 mouse fibroblasts were cultured in 75 cm^2^ tissue culture flasks in DMEM/F-12 supplemented with 10% Newborn calf serum at 37 °C in a water-saturated 5% CO_2_ atmosphere. The day before transfection 1.5–2.0 × 10^5^ cells were seeded onto 25-mm round coverslips in six-well plates and grown overnight. On the day of transfection with a 70–90% level of confluency, the cells were transfected with 0.8 μg of plasmid DNA per well using Effectene according to the manufacturer's protocol.

### Microscopy and FRAP analysis

Microscopy was performed 24–32 h post-transfection using an inverted LSM 700 confocal microscope and a Plan apochromat × 63/1.4 NA objective. The solid-state lasers at 405 and 488 nm were used for excitation. A short pass filter below 490 nm and a long pass filter beyond 490 nm were used for DAPI and mEos3.2 fluorescence, respectively. Images were acquired in 512 × 512 pixels with a 0.07 μm pixel size, 12-bit grey-scale depth, line averaging of 4 and a pixel dwell time of 6.30 μs. The laser power was set at 1.5–3.0%, the master gain at 750–800 and the digital gain at 1.5 using a pinhole size of 60.5 μm. FRAP bleaching and time-series images were acquired in 128–128 pixels with 0.07 μm pixel size, 12-bit grey-scale depth and a pixel dwell time of 1.58 μs (scan time: 121 ms). Master gain and digital gain were 750–800 and 1.5, respectively, with the pinhole set at 201 μm. A circular spot of 14 pixels (1.04 μm) in diameter was used for bleaching. Twenty prebleach images were acquired before 10 iterations of a bleaching pulse at 80% laser power used and images were acquired for the subsequent 34 s. Photobleaching during the time-series was corrected using the intensity in the bleach region relative to the entire acquisition region. The time-intensity acquisitions were normalized to the pre-bleach intensity and the first image after the bleach pulse. Results are averaged over 30–40 individual FRAP curves for the wild type and the mutants.

### Statistics

The experimental values were compared using the two tailed Student's *t*-test. In the figures, statistical significance was indicated with an asterisk if *P*<0.05.

## Additional information

**How to cite this article:** Kilic, S. *et al.* Multivalency governs HP1α association dynamics with the silent chromatin state. *Nat. Commun.* 6:7313 doi: 10.1038/ncomms8313 (2015).

## Supplementary Material

Supplementary Information Supplementary Figures 1-11, Supplementary Table 1, Supplementary Methods and Supplementary References

## Figures and Tables

**Table 1:** Kinetic parameters of HP1α - chromatin interaction dynamics.

**Experimental system**		**Dissociation kinetics**				**Association kinetics**		**Equilibrium**	**Replicates**
**Chromatin**	**Effector**	***τ***_**off,1**_ **(s)**	**% A1**	***τ***_**off,2**_ **(s)**	**% A2**	***λ***_**on**_ **(s)**	***k***_**on**_ **(M-1 s-1) × 10**^6^	***K***_**d**_ **(μM)** *	***n*** **(*****n*****′)** †
H3K9me3 (100%)	HP1α	0.25±0.03	87±7	2.26±1.22	13±7	22.9±9.8	3.64±1.56	1.16±0.54	16 (8)
H3K9me0	HP1α	0.08±0.03	83±10	(3.70±4.90)‡	17±10	N/A§	N/A§	N/A§	3 (2)
H3K9me3 (75%)	HP1α	0.19±0.01	95±3	4.03±4.09	5±3	51.0±25.8	1.63±0.82	3.23±1.63	5 (3)
H3K9me3 (50%)	HP1α	0.17±0.03	94±6	4.09±3.62	6±6	48.2±15.2	1.73±0.55	3.40±1.24	6 (4)
H3K9me3 (25%)	HP1α	0.15±0.02	95±2	4.57±3.13	5±2	75.8±23.0	1.10±0.33	6.06±1.99	6 (3)
H3K9me3 (MN)	HP1α	0.13±0.01	78±11	(1.18±0.62)‡	22±11	N/A§	N/A§	N/A§	3 (3)
H3K9me3 (100%)	HP1α+50 nM comp.	0.19±0.05	94±3	3.97±1.31	6±3	23.8±2.84	3.55±0.42	1.48±0.43	5 (3)
H3K9me3 (100%)	HP1α+250 nM comp.	0.13±0.02	93±2	3.93±1.73	7±2	43.3±15.8	2.07±0.62	3.72±1.25	3 (2)
H3K9me3 (100%)	HP1α+1000, nM comp.	0.11±0.01	93±4	2.75±0.72	7±4	29.3±5.51	2.92±0.57	3.11±0.67	4 (2)
H3K9me3 (100%)	HP1αcdm	0.33±0.01	87±1	3.40±0.53	13±1	7.45±1.87	22.4±0.6||	0.13±0.01	4 (2)
H3K9me3 (100%)	HP1α (I163E)	0.23±0.10	85±7	(4.11±2.83)‡	15±7	N/A§	N/A§	N/A§	4 (2)
H3K9me3 (100%)	HP1α (W40A)	0.10±0.02	85±21	(0.56±0.49)‡	15±21	N/A§	N/A§	N/A§	2 (2)
H3K9me3 (100%)	HP1α+P3	0.33±0.04	95±4	4.80±3.96	5±4	10.5±0.59	7.94±0.45	0.38±0.05	4 (2)
H3K9me3 (100%)	HP1α+P4	0.28±0.01	93±4	4.45±2.10	7±4	19.6±6.52	4.25±1.41	0.84±0.28	4 (3)

The percentage numbers in brackets denote the H3K9me3 modification density. MN denotes mononucleosomes, *n* is number of replicates.

^*^The *K*_d_s are calculated based on the fast kinetic phase of the dissociation process.

^†^*n* indicates the number of independent HP1α injections, *n*′ indicates the number of flow cells used.

^‡^Due to insufficient statistics, *τ*_off,2_ is poorly defined.

^§^Due to insufficient statistics we cannot accurately determine association kinetics with *λ*_on_>100 s.

^||^*k*_on_ is calculated per molecule of HP1_αcdm_.
