# Supplementary material for: Multivalency governs HP1α association dynamics with the silent chromatin state
Source: Nat Commun. 2015 Jun 18;6:7313. doi: 10.1038/ncomms8313 (PMC4557296; doi:10.1038/ncomms8313)
Supplement: Supplementary Information — Supplementary Figures 1-11, Supplementary Table 1, Supplementary Methods and Supplementary References [file ncomms8313-s1.pdf]

Supplementary Figures

Supplementary Figure 1

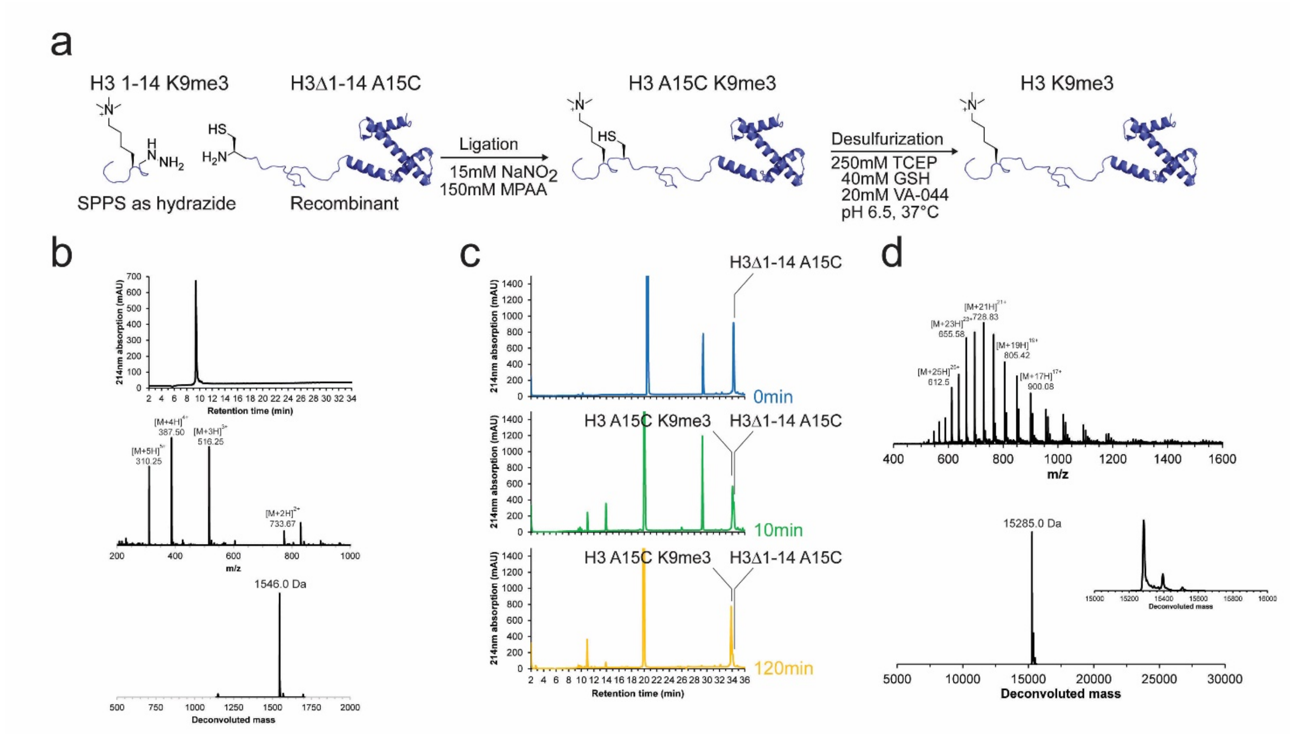

Supplementary Figure 1 | Traceless semisynthesis of H3K9me3: a) Synthesis scheme of H3K9me3: Initial oxidation of the C-terminal hydrazide in the H3(1-14)-NH-NH_2 peptide is followed by *in situ* thioester formation and ligation by mixture with a solution of MPAA and the H3(Δ1-14)A15C protein. After ligation the protein was desulfurized using a radical-initiated reaction. **b)** RP-HPLC and ESI-MS analysis of the H3(1-14)-NH-NH_2 peptide (MW calculated: 1546.8 Da, observed 1546.0 Da). **c)** Reaction progress of the ligation by RP-HPLC (gradient 0 -70% RP-HPLC buffer B in 35 min), showing the generation of the ligation product H3(A15C)K9me3 at 34 min. **d)** RP-HPLC and ESI-MS analysis of the ligation product H3K9me3 H3(A15C)K9me3 after desulfurization (MW calculated: 15283.9 Da, observed 15285.0 Da).

Supplementary Figure 2

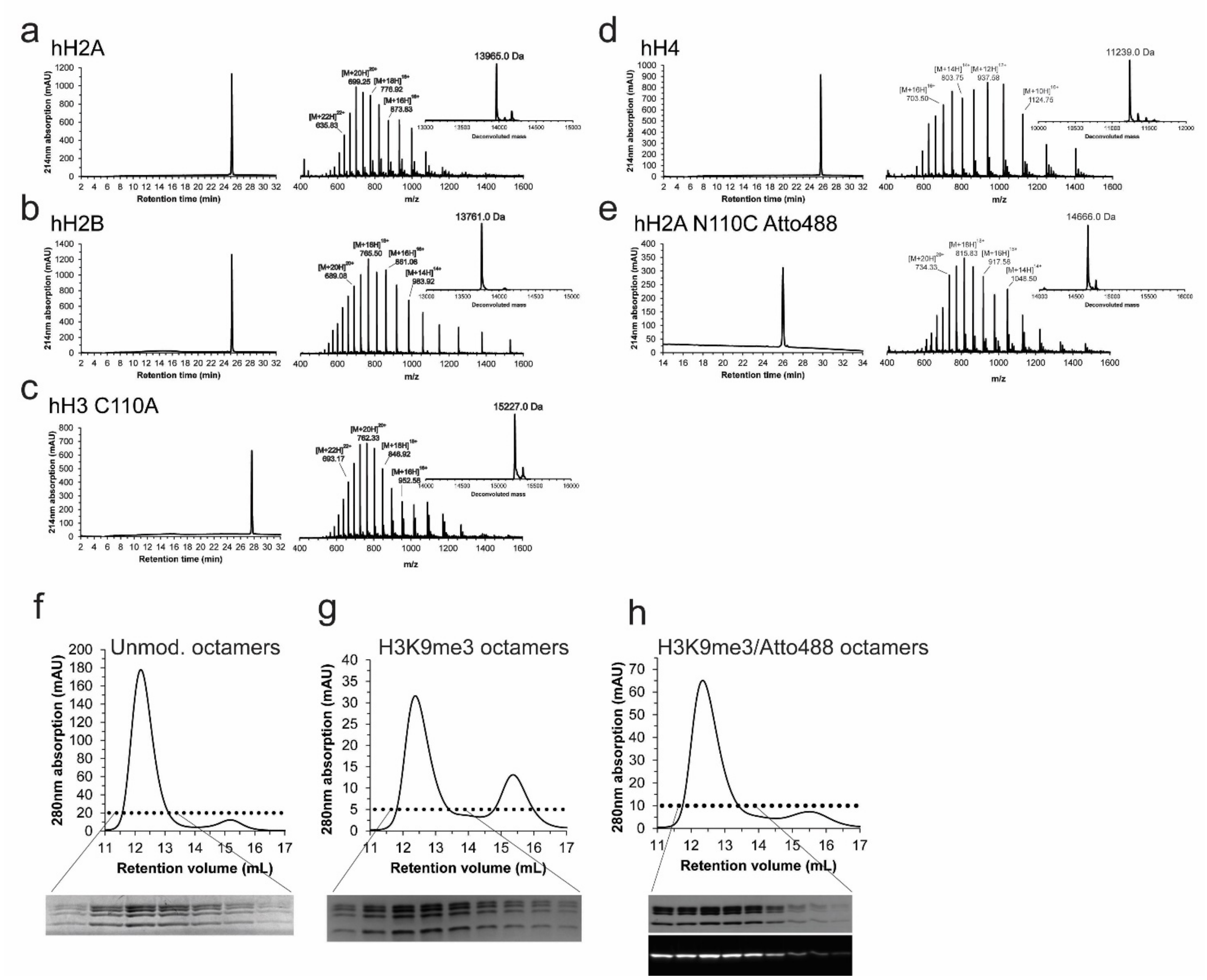

Supplementary Figure 2 | Production of human core histones and octamers. a) RP-HPLC and ESI-MS analysis of human H2A (MW calculated: 13964.26 Da, observed 13965.0 Da) **b)** RP-HPLC and ESI-MS analysis of human H2B (MW calculated: 13758.91 Da, observed 13761.0 Da) **c)** RP-HPLC and ESI-MS analysis of human H3 C110A (MW calculated: 15224.73 Da, observed 15227.0 Da), **d)** RP-HPLC and ESI-MS analysis of human H4 (MW calculated: 11236.11 Da, observed 11239.0 Da), **e)** RP-HPLC and ESI-MS analysis of H2A(Atto488) (MW calculated: 14665.3 Da, observed 14666.0 Da). **f)** Purification and analysis of unmodified histone octamers. **g)** Purification and analysis of H3K9me3 containing histone octamers. **h)** Purification and analysis of H2A(A488) histone octamers.

Supplementary Figure 3

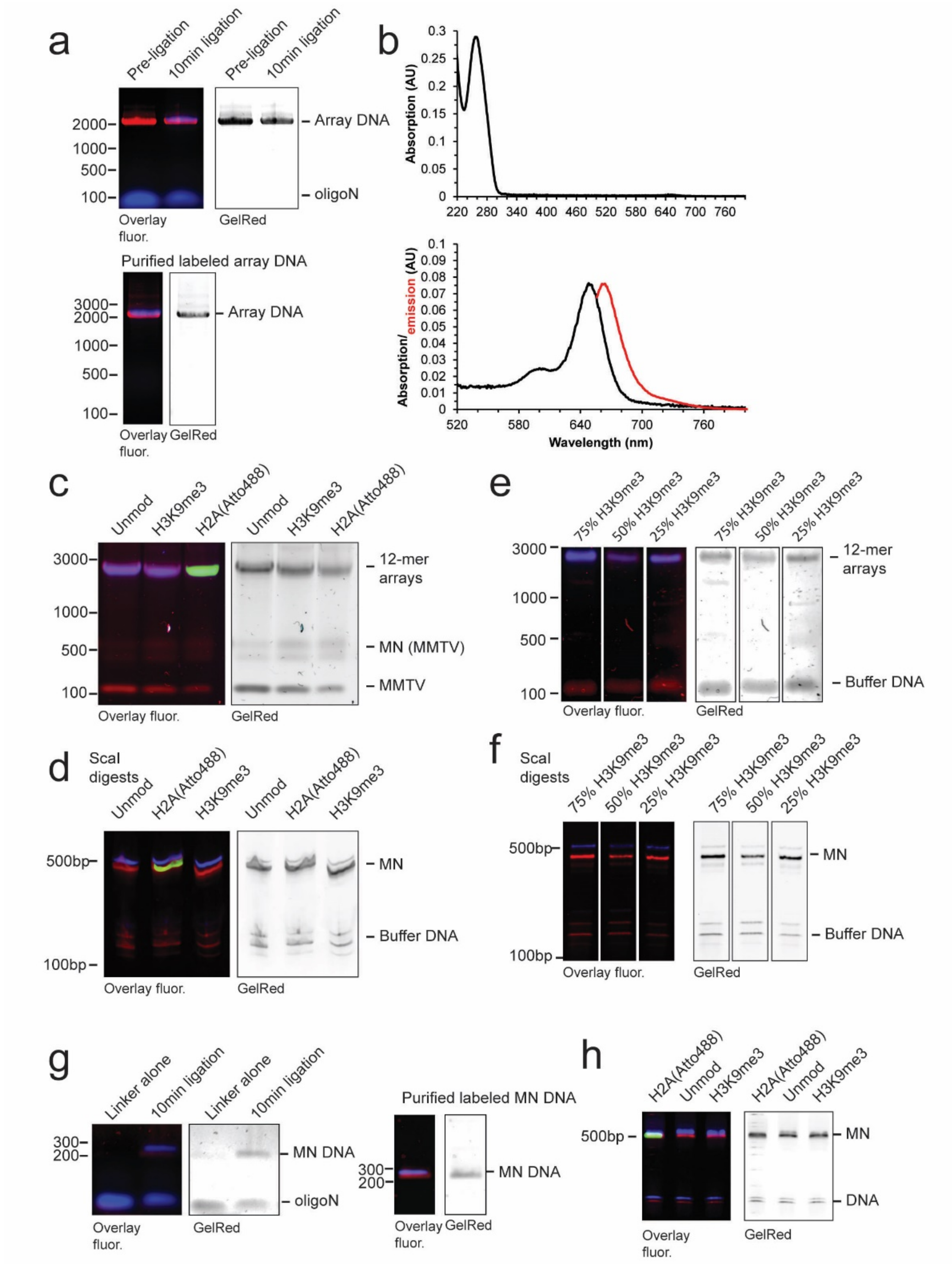

Supplementary Figure 3 | Chromatin assembly. a) Production of fluorescently labeled and biotinylated 12x601 NPS array DNA. **b)** Absorption spectrum and fluorescence emission spectrum of fluorescently labeled array DNA. **c)** Agarose gel analysis of reconstituted unmodified, H3K9me3 or Atto488 labeled chromatin arrays. **d)** Native PAGE analysis of ScaI digested (Cleaved between nucleosomes) reconstituted

arrays. **e)** Agarose gel analysis of chromatin arrays with different degrees of methylation. **f)** Native PAGE analysis of ScaI digested chromatin arrays with different levels of H3K9me3. **g)** Fluorescence labeling of mononucleosomal DNA by ligation and the isolated purified DNA. **h)** Native PAGE analysis of reconstituted mononucleosomes with different octamers.

Supplementary Figure 4

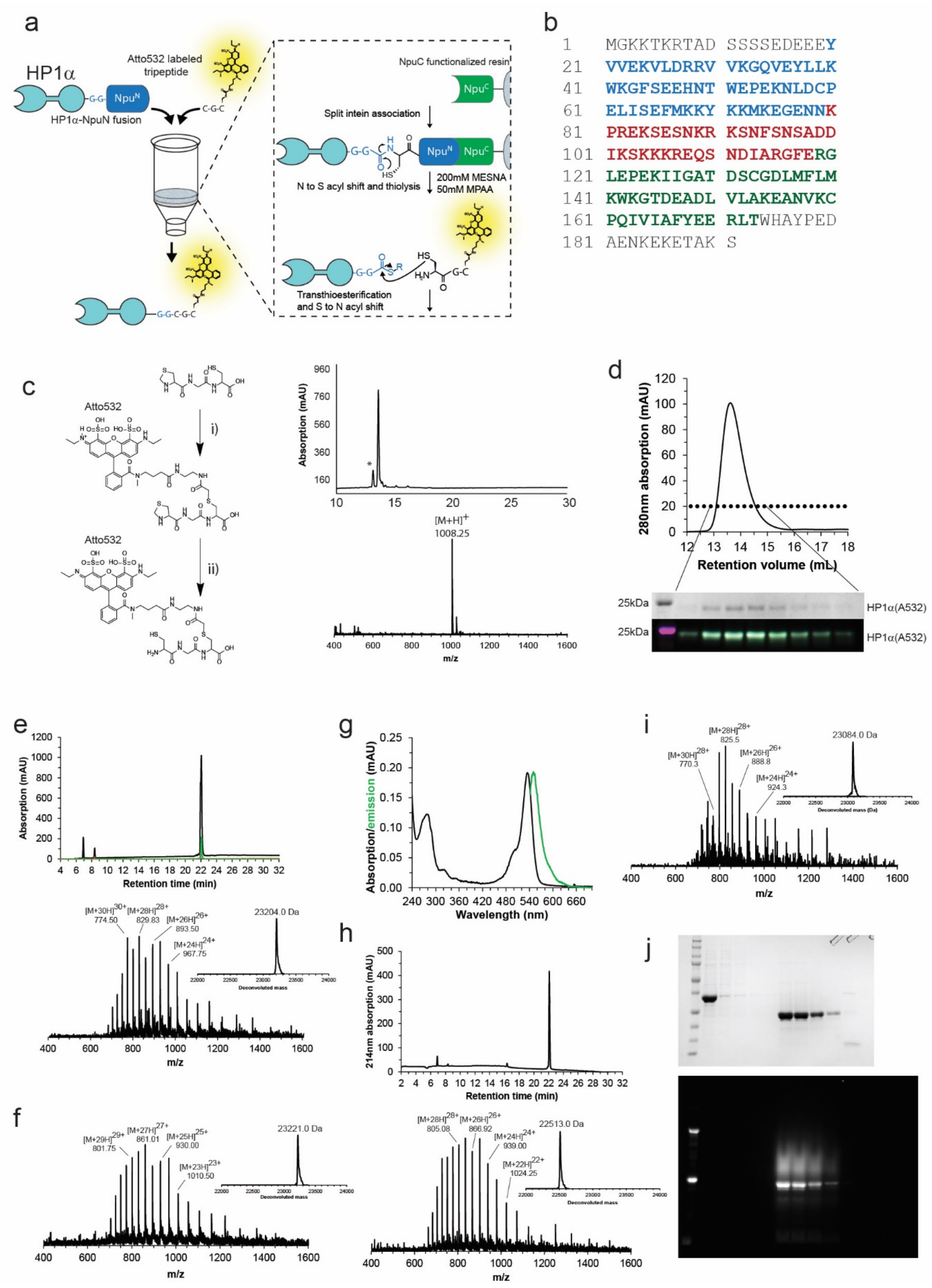

Supplementary Figure 4 | HP1α labeling & unlabeled HP1α expression. a) Scheme of fluorescent labeling of HP1α using split-intein mediated EPL. **b)** Amino acid sequence of HP1α (CD in blue, hinge in red, CSD in green). **c)** Synthesis of **P1**. After manual synthesis on the solid phase, cleavage and purification, i) **P2** is coupled to Atto532-iodoacetamide in solution (20 mM Tris, pH 7.5), followed by ii) opening of the thiazolidine using 0.5 M methoxylamine at pH 5. RP-HPLC and ESI-MS of the product are shown (MW 1009.26 Da, observed mass 1008.25 Da). **d)** Size exclusion chromatography purification of HP1α after labeling. **e)** RP-HPLC and ESI-MS analysis of labeled HP1α (MW calculated 23200.0 Da, observed MW = 23204.0 Da). **f)** ESI-MS analysis of labeled HP1α(I163E) (MW calculated 23213.0 Da, observed MW = 23221.0 Da). **g)** UV absorption spectrum and normalized fluorescence emission spectrum of Atto532 labeled HP1α. **h)** RP-HPLC and ESI-MS analysis of unlabeled HP1α (MW calculated 22506.21 Da, observed MW = 22513.0 Da). **i)** ESI-MS analysis of labeled HP1a(W40A) (MW calculated 23084.9 Da, observed MW = 23084.0 Da). **j)** Full gels of Fig. 2a (upper panel: Coomassie stain, lower panel: Atto532 fluorescence).

Supplementary Figure 5

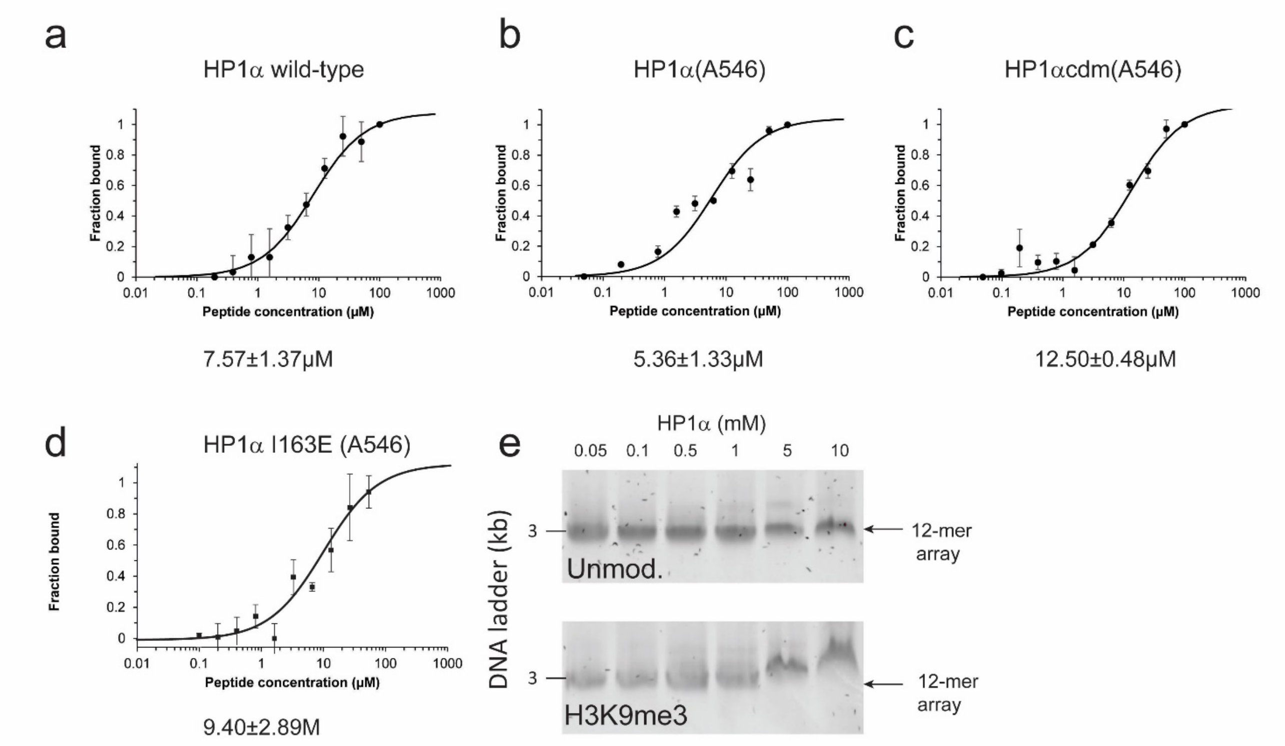

Supplementary Figure 5 | Peptide and chromatin binding of HP1α. a) HP1α binding to a H3K9me3(1-14) peptide analyzed by change of intrinsic tryptophan fluorescence and fit to the quadratic binding equation with a one-site binding model. **b)** H3K9me3 binding of HP1α(A532) analyzed by microscale thermophoresis and fit as in a). **c)** H3K9me3 binding of HP1α_{cdm} analyzed by microscale thermophoresis and fit as in a). **d)** H3K9me3 binding of HP1α(I163E) analyzed by microscale thermophoresis and fit as in a) **e)** EMAC analysis of HP1α binding to unmodified or H3K9me3 containing 12-mer chromatin arrays. No binding is detected for the unmodified arrays up to 10 μM HP1α, whereas a significant gel-shift indicates specific H3K9me3 recognition in the modified arrays.

Supplementary Figure 6

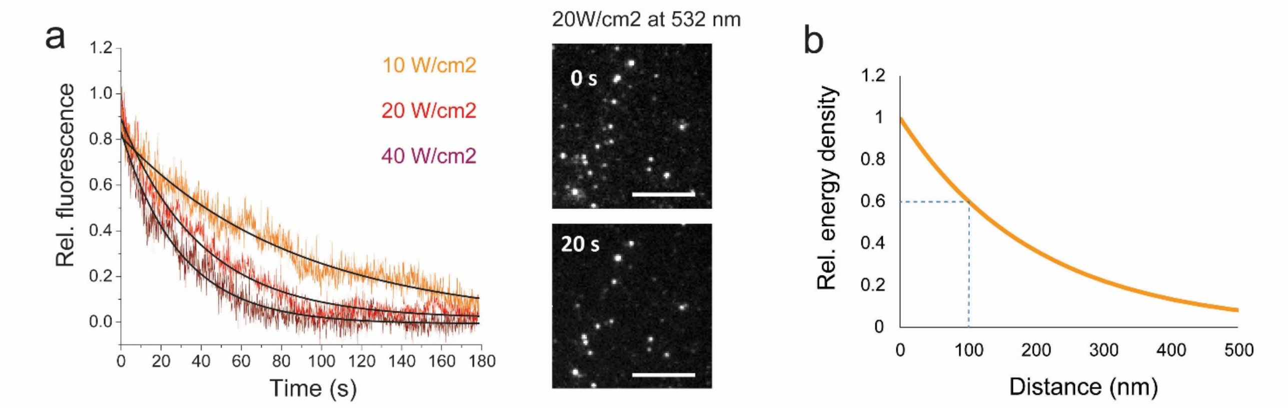

Supplementary Figure 6 | Photobleaching kinetics of HP1α(Atto532) and laser penetration under TIRF conditions. a) HP1α was biotinylated using EZ-link biotin (Pierce) and immobilized on PEG-passivated coverslips using neutravidin coupling. Single HP1 molecules were imaged (inset at the right at 0 and 20s of irradiation) at different intensities of laser irradiation (10-40 W/cm2) and photobleaching kinetics were determined for 200-300 molecules. The accumulated kinetics were fitted to single-exponential decay, resulting in bleaching kinetics of τ_{bleach} = 29.6 s for 40 W/cm2, τ_{bleach} = 40.0 s for 20 W/cm2 and τ_{bleach} = 86.6 s for 10 W/cm2. Scale bars: 5 μm. **b)** Measured laser penetration at 532 nm under TIRF conditions. The dashed lines indicate the approximate length of a stretched 12-mer chromatin array, demonstrating up to 40% variation in emission intensity for HP1α bound to chromatin, depending on the binding site in the chromatin array.

Supplementary Figure 7

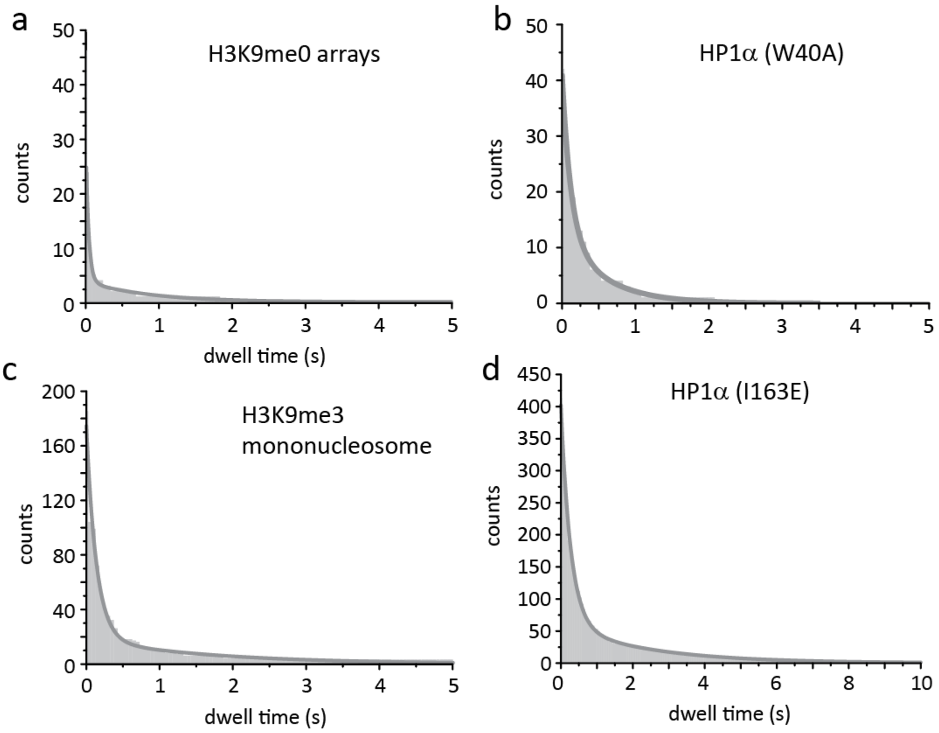

Supplementary Figure 7 | HP1α interaction with unmodified arrays and mononucleosomes, and HP1α (I163E) mutant. a) HP1α binding to unmodified arrays: Very few events are detected and measured events decay with $\tau_{off,1}$ = 0.08 ± 0.04 s $\tau_{off,2}$ = 3.88 + 4.90 s**. b)** HP1α(W40A) mutant analysis: A fit to the cumulative histogram of on-times reveals $\tau_{off,1}$ = 0.10 ± 0.20 s and $\tau_{off,2}$ = 0..28 ± 0.39 s. **c)** HP1α binding to mononucleosomes carrying H3K9me3: A fit to the cumulative histogram of on-times reveals $\tau_{off,1}$ = 0.13 ± 0.01 s and $\tau_{off,2}$ = 1.18 ± 0.62 s. **c)** HP1α(I163E) dimerization mutant analysis: A fit to the cumulative histogram of on-times reveals $\tau_{off,1}$ = 0.23 ± 0.10 s and $\tau_{off,2}$ = 4.11 ± 2.83 s. For all histograms, 100 chromatin arrays were analyzed (105 frames each).

Supplementary Figure 8

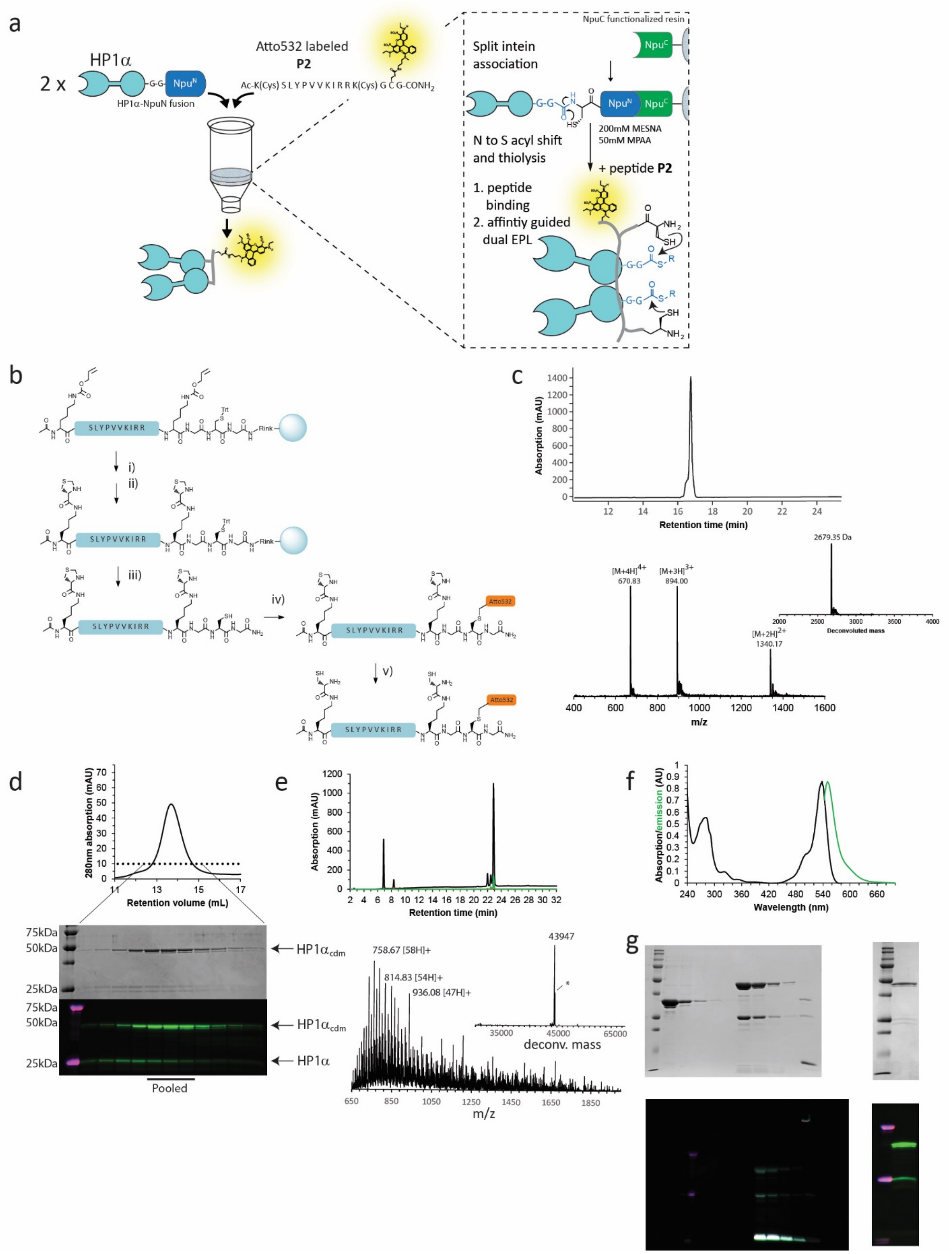

Supplementary Figure 8 | Synthesis of HP1α_{cdm}. a) Scheme of HP1α_{cdm} synthesis using an affinity guided dual EPL strategy. **b)** Synthesis scheme for production of **P2**. i) On-resin deprotection of alloc-K using $Pd(PPh_3)_4$ / $PhSiH_3$ in DCM, ii) coupling of Boc-Thz using HBTU/HOBt in DMF, iii) peptide cleavage using TFA/TIS/H2O 95:2.5:2.5, iv) labeling with Atto532-iodoacetamide in 200 mM phosphate, pH 7.3, 5M GdmHCl, v) opening of Thz using 0.5 M methoxylamine at pH 5. **c)** RP-HPLC and MS analysis of **P2** (MW calculated: 2680.24 Da, measured 2679.35 Da). **d)** Size exclusion chromatography purification of HP1α_{cdm}, and SDS-PAGE analysis of the fractions. The pooled fractions containing only HP1α_{cdm} are indicated. **e)** RP-HPLC and MS analysis of the final product, HP1α_{cdm} (calculated mass: 43944.65 Da, observed mass 43947.0, *: buffer adduct (HEPES/Na+)). **f)** UV absorption spectrum and normalized fluorescence emission spectrum of HP1α_{cdm}. **g**) Full gels of Fig. 3b (upper panels: Coomassie stain, lower panels: Atto532 fluorescence).

Supplementary Figure 9

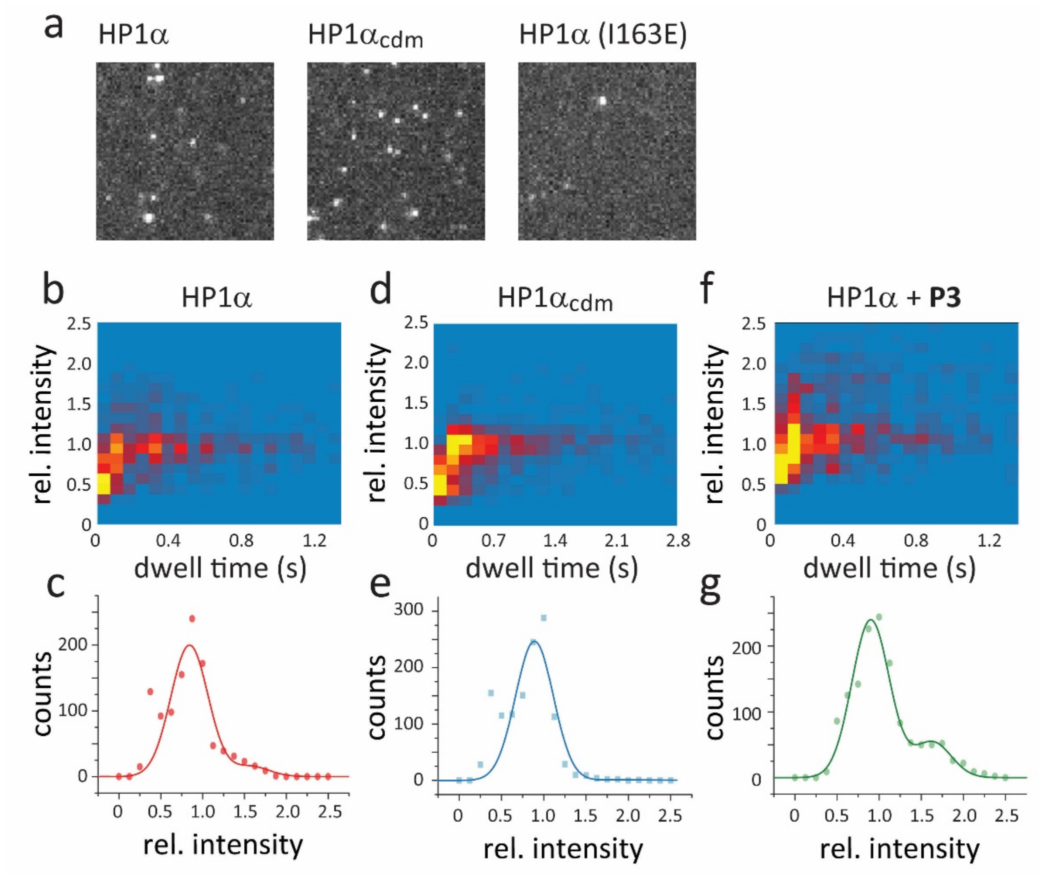

Supplementary Figure 9 | Analysis of signal intensities in HP1α binding experiments. a) Fluorescent background of HP1α, HP1αcdm and HP1α(I13E) experiments: The background mainly arises from diffusing labeled proteins, similar background values are measured, indicating that HP1α concentrations are comparable between the experiments. **b, d, f)** Two-dimensional histogram of the intensity (normalized to emission of a single Atto532 dye) versus the duration of individual observations for the indicated proteins. **c, e, g)** histogram of the intensity of individual observations for the indicated proteins. The solid lines are fits to a double Gaussian function, describing the expected peak distribution for a given percentage of dimeric HP1α at a set labeling efficiency. Assuming protein labeling of 50% (60% in the initial preparation as determined by UV, including photobleaching) dimerization can be quantified as 17% for HP1α and 42% for HP1α + **P3**.

Supplementary Figure 10

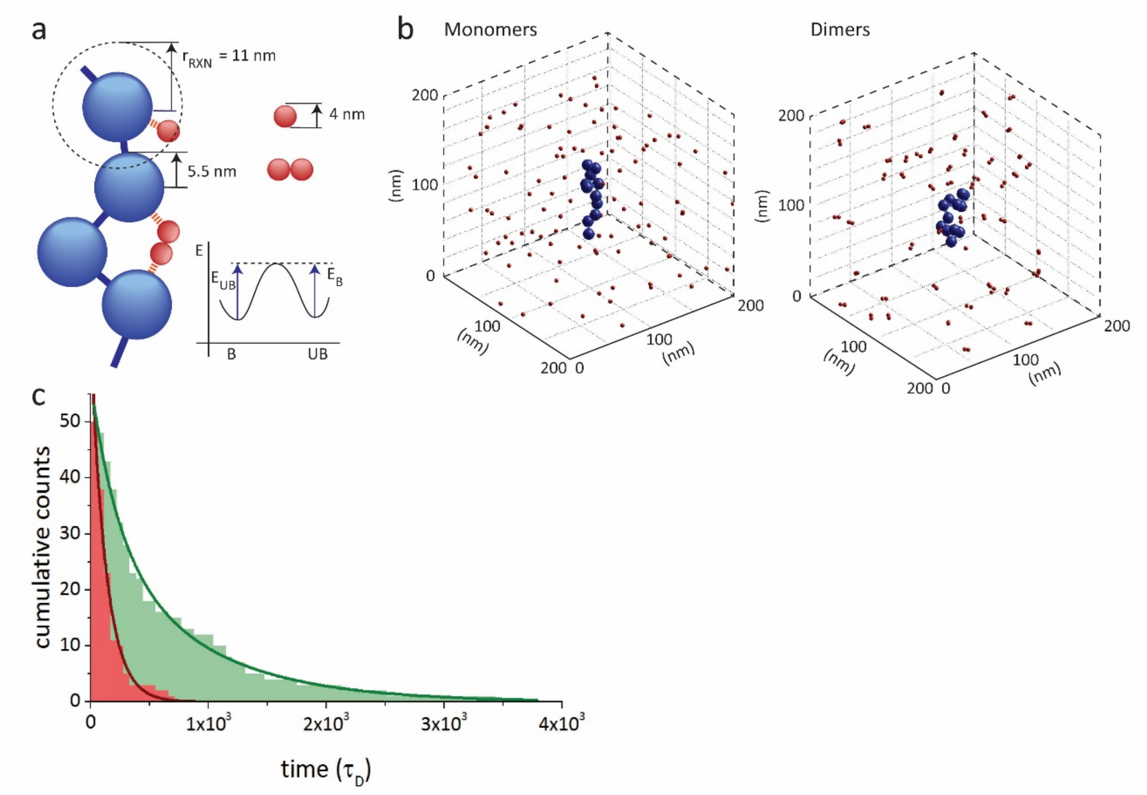

Supplementary Figure 10 | Brownian dynamics simulation of monomeric and dimeric binder interaction with chromatin chains. a) Setup of the model: Chromatin is represented by a string of spheres with a diameter of 5.5 nm corresponding to individual nucleosomes. Binders (HP1α) are represented by spheres with 2 nm radius and are either monomeric, or dimeric (using a harmonic potential to enact dimerization). If within a radius of 11 nm (nucleosome diameter + range of the H3 peptide tail), binders can interact with nucleosomes. The interaction is determined using a Monte-Carlo process, based on an energy landscape description of the binding reaction and using an energy barrier E_B for binding and E_{UB} for dissociation. Bound factors interact with nucleosomes through a harmonic potential, until released. Each nucleosome can only interact with one binder at a time. **b)** Initial conditions for simulations for monomeric or dimeric binders: 200 monomers / 100 dimers are placed at random into a 200 nm x 200 nm x 200 nm box containing a randomly generated 12-nucleosome array. After initial equilibration, the first-passage time for a binding event is simulated. Then, the simulation is reset and the process is repeated until sufficient

statistics are generated. **d)** Dissociation kinetics of bound factors: Monomeric factors (red) exhibit single exponential release kinetics with fitted time constant of $\tau_{off} = 130\ \tau_D$. Dimeric factors exhibit double exponential dissociation kinetics due to the possibility of multivalent engagement of two nucleosomes (low probability). Fitted time constants are $\tau_{1,off} = 228\ \tau_D$ and $\tau_{2,off} = 845\ \tau_D$.

Supplementary Figure 11

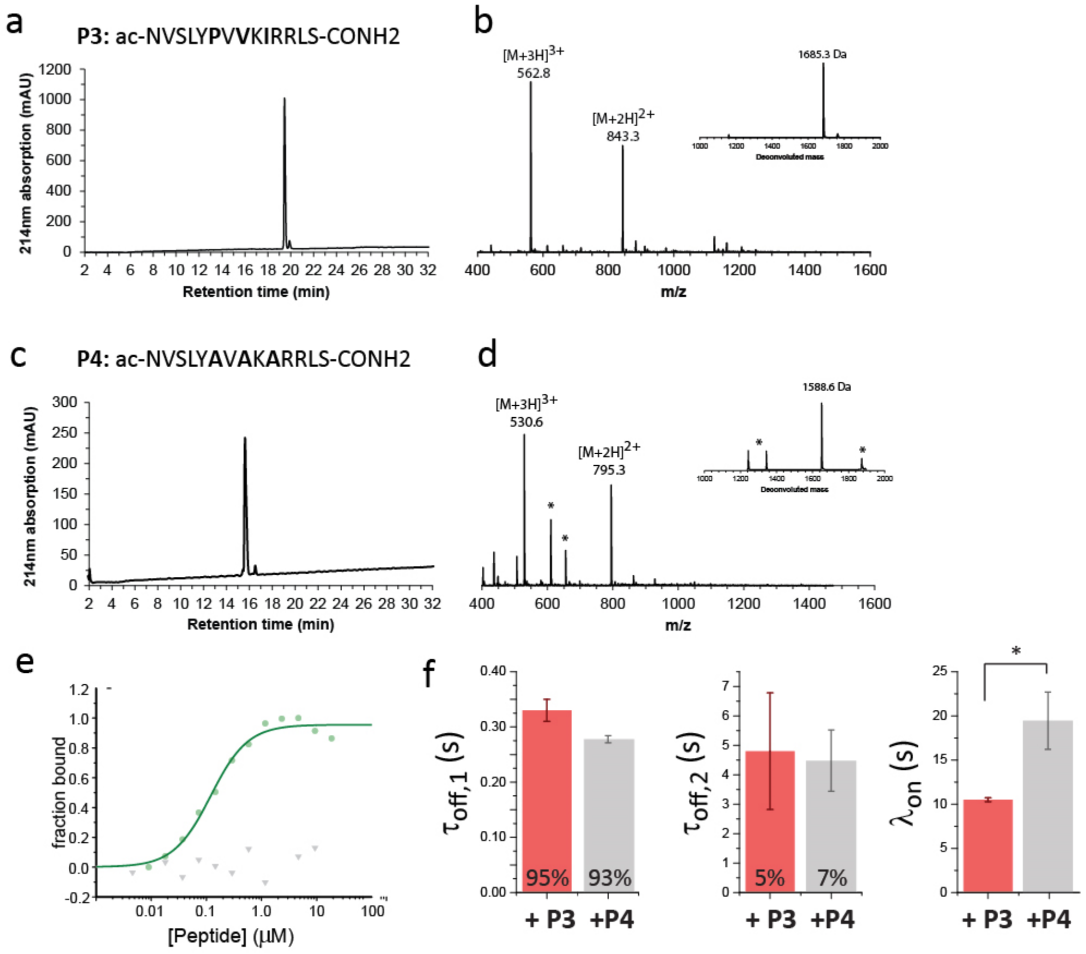

Supplementary Figure 11 | Synthesis and analysis of P3 and P4. a) Sequence of **P3**, HPLC and **b)** ESI-MS analysis of purified **P3,** (calculated mass: 1685.03 Da, observed mass 1685.3 Da). **c)** Sequence of **P4**, HPLC and **d)** ESI-MS analysis of purified **P4,** (calculated mass: 1589.03 Da, observed mass 1589.6 Da). **e)** Microscale thermophoresis analysis of binding of **P3** (green) and **P4** (grey) to HP1α, labeled with Atto532. The solid line is a fit to a one-site equilibrium binding model and results in a K_D = 0.11 μM. **f)** Comparison of the average dissociation time constants $\tau_{off,1}$ and $\tau_{off,2}$ (fast and slow phase) between HP1α in the presence of **P3** and **P4** (error bars: S.E.M., n = 4,2 replicates, *: $p<0.05$, student's t-test). Comparison of the association rate constant k_{on} between HP1α in the presence of **P3** or **P4** (errors: SEM., n = 4 replicates, *: $p<0.05$, student's t-test).

Supplementary Tables

Supplementary Table 1

Parameters for kinetic model (Fig. 4f)

Parameter	Value	Note
kassoc	2.87 x 108 s-1	
k'assoc	2.31 x 108 s-1	adjusted for diffusion coefficient of dimeric HP1
kdissoc	3.35 x 103 s-1	nonspecific Kd: 11.6 mM
α	5	increased residence time of dimeric HP1
kbind	27 s-1	
krelease	8.7 s-1	residence time of 115 ms in the absence of rapid rebinding
kbiv	0.025 s-1	in exteded fibers, second binding event is sterically hindered
kdim	1.0 x 106 s-1	diffusion controlled
kmono	1.0 x 10-1 s-1	Kd of HP1 dimerization: 0.1 mM

Supplementary Methods

Materials

Amino acid derivatives and resins were purchased from Novabiochem, Merck (Hohenbrunn, Germany). Peptide synthesis solvents and reagents were from Acros Organics (Geel, Belgium) (Dimethylformamide, dichloromethane, N,N-diisopropylethylamine and piperidine) or Protein Technologies Inc. (Tucson, AZ, US) (HBTU). All commonly used chemical reagents and solvents were purchased from Sigma-Aldrich Chemical Company (Steinheim, Germany), Fischer Scientific (Fair lawn, NJ, US/Loughborough, UK) or Applichem (Darmstadt, Germany). Acetonitrile was from Lab-Scan Analytical Sciences (Sowinskiego, Poland). Chemically competent DH5a, BL21(DE3) and BL21(DE3)plysS were from Novagen (Darmstadt, Germany) and used to generate in-house stocks of competent cells. T4 DNA ligase, restriction enzymes (EcorV-HF, ScaI-HF, BsaI-HF, BglII-HF, and PstI-HF, Xba, BamHI-HF), Phusion DNA polymerase, DNA ladders, DNA loading dyes and dNTPs were purchased from New England Biolabs (Ipswich, MA, US) distributed through BioConcept (Allschwil, Switzerland). Primers were ordered from and synthesized by Integrated DNA technologies (Leuwen, Belgium) or Microsynth (Balgach, Switzerland). Gene sequencing was performed at Microsynth (Balgach, Switzerland) or GATC Biotech (Constance, Germany). Single-point mutations were done using a QuikChange II XL site-directed mutagenesis kit from Agilent (Basel, Switzerland). Atto488 maleimide, Atto532 iodoacetamide and Atto647N NHS ester were purchased from ATTO-TEC Gmbh (Siegen, Germany). Materials for hand-casting of agarose and SDS-PAGE gels (agarose, TEMED, APS, acrylamide), analysis of SDS-PAGE gels (Precision Plus Protein™ All blue standards and Precision Plus Protein™ dual color standards) and pre-cast Criterion 5% TBE gels were purchased from BioRad (Hercules, CA, US). QiaQuick spin column for PCR purification, gel extraction and nucleotide removal as well as QiaPrep spin columns for miniprep plasmid purification were from Qiagen (Hilden, Germany). Slide-a-lyzer dialysis casettes and MINI dialysis buttons were from Thermo Scientific (Rockford, IL, US). Amicon Ultra

centrifugal concentration units were from Merck Millipore (Tullagreen, Ireland). Spectra/por dialysis tubing was from Spectrum laboratories Inc (Rancho Dominguez, CA, US).

Equipment

Bacterial cells for recombinant protein expression were grown in an HT infors AG incubator, recovered with an Avanti J-20 XPI centrifuge from Beckman Coulter and lysed by freeze-thawing and sonication using a Vibra-cell VCX 750 Sonics & Materials sonicator. Manual peptide synthesis and reactions on solid-phase were carried out in reaction vessels from Peptides International and automated peptide synthesis done on a Tribute instrument from Peptides International Inc. Size exclusion chromatography and ion exchange purification was performed on an AKTA Pure FPLC system from GE Healthcare. Size-exclusion was done using an S200 10/300GL column from GE Healthcare using isocratic elution with a flow rate of 0.5mL/min over 1.05 column volumes and collected as 250µL fractions in 96-well plates. Cation exchange and anion exchange purification was done using HiTrap SP HP (5mL) and HiTrap Q FF (1mL) traps from GE Healthcare. Analytical reversed-phase HPLC (RP-HPLC) was performed on an Agilent 1260 series instrument with an Agilent Zorbax C18 column (5µm, 4.6 x 150 mm), employing 0.1% TFA in water (RP-HPLC solvent A), and 90% acetonitrile, 0.1% TFA in water (RP-HPLC solvent B), as the mobile phases. Typical analytical gradients were 0-70% solvent B over 30 min at a flow rate of 1 mL/min. Preparative scale purifications were conducted on an Agilent 1260 preparative HPLC system. A Zorbax C18 preparative column (7 µm, 21.2 x 250 mm) or a semi-preparative column (5µm, 9.4 x 250 mm) was employed at a flow rate of 20 mL/min or 4 mL/min, respectively. ESI-MS analysis was done using a LCQFleet Ion trap Thermo Fisher Mass spectrometer. Absorbance spectra were recorded with an Agilent 8453 UV-Vis spectrophotometer. Fluorescence spectra and tryptophan fluorescence titration was carried out with a Fluorolog®-3 Horiba Jobin Yvon spectrofluorometer. SDS-PAGE, native PAGE and agarose gels were imaged using a ChemiDoc MP imaging system from BioRad. Titrations using microscale thermophoresis were done on a MonoLith NT.115 instrument equipped with blue/green or green/red filters from NanoTemper technologies. Live-cell

confocal microscopy was done on a LSM700 inverted microscope from Zeiss. For single-molecule TIRF microscopy a Nikon Ti-E inverted fluorescence microscope, controlled by NIS-elements, equipped with a CFI Apo TIRF 100x Oil immersion objective (NA 1.49) was used. Further specifications of the microscope are detailed under smTIRFM imaging.

Preparation of designer chromatin

Expression and purification of recombinant human histones

Human *wild-type* histones were prepared as described in ref. [1] with minor alterations. The histones were expressed in BL21 DE3 plysS cells from genes inserted into a pet15b plasmid. Cell cultures were grown at 37°C in LB media supplemented with 100µg/mL ampicillin and 35µg/mL chloramphenicol until an OD600 of 0.6. Protein expression was induced by addition of IPTG to a final concentration of 0.5mM. Expression of the protein was allowed to continue until 3h post-induction. Cells were harvested, cell pellets were resuspended in lysis buffer (20mM Tris, 1mM EDTA, 200mM NaCl, 1mM βME, 1 protease inhibitor tablet/50mL, pH 7.5). Cells were lysed by freeze-thawing and sonication. The inclusion body pellet was washed twice with 7.5mL of histone lysis buffer with 1% triton and once without triton. Histones were resolubilized in histone resolubilization buffer (6M GdmCl, 20mM Tris, 1mM EDTA, 1mM βME, pH 7.5), dialyzed into urea buffer (7M urea, 10mM Tris, 1mM EDTA, 0.1M NaCl, 5mM βME, pH 7.5) followed by purification by cation exchange (using a HiTrap SP HP 5mL column). Collected fractions were analyzed by SDS-PAGE, followed by final purification using preparative RP-HPLC. Collected fractions were characterized by analytical RP-HPLC and ESI-MS (**Supplementary Fig. 2a-d**), lyophilized and stored at -20°C until use in octamer refolding. H2A: Calculated MW = 13964.26 Da, observed MW = 13965.0 Da. H2B: Calculated MW = 13758.91 Da, observed MW = 13761.0 Da. H3 C110A: Calculated MW = 15224.73 Da, observed MW = 15227.0 Da. H4: MW = 11236.11 Da, observed MW = 11239.0 Da.

Labeling of H2A N110C with Atto488-maleimide

In a typical reaction, 2 mg HPLC purified H2A N110C (0.15 μmol) was dissolved in 250 μL labeling buffer (20 mM Tris, pH 7.8, 6 M guanidinium hydrochloride, 200 μM TCEP) and 0.5 mg Atto488-maleimide (0.5 μmol, 3.3 equiv.) was added in 50 μl N,N-dimethylformamide (DMF) in the dark. The mixture was stirred for 1 h and quenched by addition of 1 mM β-mercaptoethanol. Atto488 labeled H2A N110C (H2A(Atto488)) was subsequently purified by semi preparative C-18 RP-HPLC with a gradient of 30-70% HPLC solvent B over 30 min. The protein was characterized by analytical HPLC and ESI-MS (Calculated MW = 14665.3 Da, observed MW = 14666.0 Da) (**Supplementary Fig. 2e**).

Synthesis of H3K9me3(1-14)

Preparation of the hydrazide resin was performed as in ref. [2], with minor alterations. 0.85 mmol of chlorotrityl resin was swollen in DMF for 15 min, and cooled to 0° C. 3 equivalents of DIEA and 2 equivalents of hydrazine monohydrate were mixed in DMF and added dropwise to the swollen resin. Then, the resin was stirred for 1 h and allowed to warm to room temperature (RT). After 1 h, 100 μL methanol was added and stirred for another 10 min at RT followed by a DMF wash. The first amino acid (Lys) was coupled manually, following standard synthesis protocols for a fluorenylmethoxycarbonyl (FMOC) Nα protection strategy. The resin substitution was then determined by UV quantification of released Fmoc after piperidine treatment of a defined amount of resin. The degree of substitution was typically 0.4-0.7 mmol/g. H3K9me3(1-14) was then synthesized using a Tribute automated peptide synthesizer using a standard FMOC synthesis protocol with 2-(1H-benzotriazole-1-yl)-1,1,3,3-tetramethyluronium hexafluorophosphate (HBTU) amino-acid activation. The following side-chain protection groups were used: Arg(pbf), Lys(Boc), Thr(tBu), Ser(tBu) and Gln(trt). Kme3 was introduced using the pre-methylated building

block FMOC-Lys(me3)-OH. After the synthesis, the peptide was cleaved using a cocktail of 95% trifluoracetic acid (TFA), 2.5% triisopropylsilane (TIS) and 2.5% water. Subsequently, 88 mg of the crude H3(1-14)-$NHNH_2$ peptide was purified by RP-HPLC on a preparative scale using a 0-10% gradient over 30 min, yielding 37 mg pure peptide. The H3K9me3(1-14)-$NHNH_2$ hydrazide peptide was characterized by ESI-MS (Calculated MW = 1546.8 Da, observed MW = 1546.0 Da) (**Supplementary Fig. 1b**).

Expression, cleavage and purification to generate H3 (Δ1-14) A15C

H3 Δ1-14 A15C was expressed as an N-terminal fusion to SUMO, followed by protease cleavage using SUMO protease and HPLC purification as described in ref. [3]. The protein was characterized by analytical HPLC and ESI-MS (Calculated MW = 13769.11 Da, observed MW = 13770.0 Da) (Data not shown).

Preparation of HP1α proteins

Expression and initial purification of 6xH-Thr-HP1α, HP1α-GG-NpuN-6xH, HP1α(1-176)-GG-NpuN-6xH and HP1α(I163E)-GG-NpuN-6xH, HP1α(W40A)-GG-NpuN-6xH

HP1α (CBX5) was cloned into a pET15b expression vector. For generation of labeled HP1α, the sequence of the NpuN split intein[4] was fused to the C-terminus after a double glycine linker, followed by a final hexahistidine tag (HP1α-GG-NpuN-6xH). For the production of HP1α_{cdm}, the first 176 residues of HP1α were fused to NpuN followed by a hexahistidine tag (HP1α(1-176)-GG-NpuN-6xH). For HP1α(I163E)-GG-NpuN-6xH and HP1α(W40A)-GG-NpuN-6xH the I to E mutation, as well as the W to A mutation were introduced by Quickchange XL II mutagenesis (Agilent). All proteins were expressed in *E. coli* BL21 DE3 cells, as follows: 3 L LB media supplemented with 100 µg/mL ampicillin were inoculated from starter cultures and incubated at 37° C with 220 rpm rotation for 2 h. The temperature was then reduced to 18° C and the cultures allowed to grow until reaching an OD600 of 0.6. Expression was induced by addition of IPTG to a

final concentration of 0.25 mM, followed by further incubation at 18° C for 16-20 h. Cells were harvested, resuspended and lysed in Ni-NTA lysis buffer (25 mM phosphate, 50 mM NaCl, 5 mM imidazole, 1 protease inhibitor tablet/50 mL, pH 8.0) by sonication. Lysed cells were centrifuged at 15000 x g for 15 min and the soluble fraction was loaded onto 2 mL of Ni-NTA resin (Qiagen) per liter culture. The protein was bound to the resin by gentle nutating for 30 min before the flowthrough was collected. Then, the resin was washed with 2x5 CV of Ni-NTA wash buffer (25 mM phosphate, 50 mM NaCl, 20 mM imidazole, pH 8.0), and the protein eluted with 2 x 1.5 CV of Ni-NTA elution buffer (25 mM phosphate, 50 mM NaCl, 400 mM imidazole, pH 8.0). Elutions were pooled and purified using anion exchange chromatography (AIEX) using a 1 mL HiTrap Q FF (GE Healthcare): After loading the protein solution, the column was washed with 3 CV of AIEX buffer A (50 mM phosphate, 50 mM NaCl, pH 7.5) and subsequently eluted using a 0-100% gradient from AIEX buffer A to AIEX buffer B (50 mM phosphate, 1000 mM NaCl, pH 7.5) over 20CV followed by 3CV of AIEX buffer B. Fractions were analyzed using SDS-PAGE analysis and pooled accordingly. For unlabeled HP1α, 10 U of thrombin (Calbiochem) were added to the pooled fractions from AIEX to remove the N-terminal 6 x His-tag. Cleavage was monitored by RP-HPLC and MS, and was typically complete after 2-3 h. The cleaved protein was then purified by gel filtration on a Superdex 200 10/300GL column, using HP1 gel filtration buffer (50 mM HEPES, 150 mM NaCl, 2.5 mM DTT, pH 7.5). Fractions containing the protein were pooled and concentrated to 40-100 µM. Then the protein was either mixed with glycerol to a final concentration of 30% glycerol, flash frozen and stored at -80° C or kept on ice at 4° C until use.

Synthesis of peptides P1, P2, P3 and P4

Peptide **P1** (Thz-G_2-C_3-$CONH_2$, Thz: thiazolidine) was synthesized manually on a Rink amide resin (Novabiochem) using Boc-Thiazolidine and FMOC-Cys(Trt)-OH, employing a FMOC-protection strategy and standard HBTU coupling protocols. The peptide was cleaved from the resin using 95% TFA, 2.5% TIS, 2.5% water and purified using preparative RP-HPLC (on a 20 - 50% B gradient). Subsequently, 1.5 mg peptide (5

μmol) was dissolved in labeling buffer (200 mM phosphate pH 7.3, 5M guanidinium hydrochloride) to a concentration of 10 mM. 1 mg of Atto532-iodoacetamide (1 μmol) was subsequently added in 50 μL DMSO, and the reaction was incubated until completed as judged by RP-HPLC. Residual unreacted dye was quenched by the addition of 2 mM β-mercaptoethanol. Then, 0.5 M methoxylamine was added and the pH was adjusted to 5 using 2 M NaOH. Opening of the thiazolidine was followed by RP-HPLC and ESI-MS. After completion the peptide was purified using semipreparative RP-HPLC and a gradient of 0-70% B in 45 min. Final products were analyzed by RP-HPLC and ESI-MS (Calculated exact mass = 1009.26 Da, observed mass = 1008.25 Da) (**Supplementary Fig. 4c**).

Peptide **P2** (ac-$K_1S_2L_3Y_4P_5V_6V_7K_8I_9R_{10}R_{11}K_{12}G_{13}C_{14}G_{15}$-$CONH_2$) was synthesized on the Tribute automated peptide synthesizer (PTI) using standard HBTU coupling protocols, and the following protecting groups (Cys(Trt), Lys(Boc), Arg(pbf), Y(tBu), S(tBu)) (See scheme in **Supplementary Fig. 8b**) . In addition, K_1 and K_{12} were included containing an Alloc-protecting group. After synthesis, the N-terminus was acetylated using 3 x 20 min incubation of the peptidyl-resin with 10% acetic anhydride, 10% *N,N*-diisopropyethylamine (DIPEA) in dimethylformamide (DMF), followed by extensive washes. Then, the alloc-groups on the two lysines were removed by 3 x 30 min treatment of the peptidyl-resin with 0.25 equivalents of Tetrakis(triphenylphosphine) palladium(0) ($Pd(PPh_3)_4$) in dichloromethane (DCM) and containing 24 equivalents of phenylsilane ($PhSiH_3$). The resin was subsequently washed with 0.5 % DIPEA in DMF, 0.5 % sodium diethylthio-carbamate in DMF, 0.5 % Hydroxybenzotriazole in DMF and finally DMF. Then, Boc-Thz was coupled to both deprotected lysines in one step using standard HBTU activation and 10 x excess to the peptide. Subsequently, the peptide was cleaved from the resin and labeled with Atto532-iodoacetamide as described for **P2**. The final product was analyzed by RP-HPLC and ESI-MS (Calculated MW = 2680.24 Da, observed MW = 2679.35 Da) (**Supplementary Fig. 8c**).

Peptide **P3** (NH_2-$N_1V_2S_3L_4Y_5P_6V_7V_8K_9I_{10}R_{11}R_{12}L_{13}S_{14}$-$CONH_2$) was synthesized on the Tribute automated peptide synthesizer (PTI) using standard HBTU coupling protocols, and the following protecting groups

(Asn(Trt), Lys(Boc), Arg (pbf), Y(tBu), Ser(tBu)). After cleavage from the resin the peptide was purified using preparative RP-HPLC and analyzed by RP-HPLC and ESI-MS (Calculated MW = 1685.03 Da, observed MW = 1685.3 Da) (**Supplementary Fig. 11**).

Peptide **P4** (NH_2-$N_1V_2S_3L_4Y_5A_6V_7A_8K_9A_{10}R_{11}R_{12}L_{13}S_{14}$-$CONH_2$) was synthesized on the Tribute automated peptide synthesizer (PTI) using standard HBTU coupling protocols, and the following protecting groups (Asn(Trt), Lys(Boc), Arg (pbf), Y(tBu), Ser(tBu)). After cleavage from the resin the peptide was purified using preparative RP-HPLC and analyzed by RP-HPLC and ESI-MS (Calculated MW = 1589.03 Da, observed MW = 1588.6 Da) (**Supplementary Fig. 11**).

Labeling of HP1α with split intein column and covalent dimerization of HP1α with split intein column

Lyophilized stocks of NpuC-peptide (prepared as decribed [4]) were dissolved in water to a concentration of 2mM, as quantified by UV spectroscopy ($\varepsilon_{280nm,NpuC\text{-}CysOMe}$ = 2980 $M^{-1}cm^{-1}$). 125 µL stocks of this were flash frozen and stored at -80°C for later use. For the preparation of NpuC-resin, 500 µL of SulfoLink resin slurry (Pierce) was put into a small fritted column and washed with water. A 125 µL stock of the NpuC-CysOMe peptide was mixed with 125 µL of 2x coupling buffer (100 mM Tris, 10 mM EDTA, pH 8.5), TCEP was added to a final concentration of 25 mM and the pH was adjusted to 8.5. The peptide solution was then added to the SulfoLink resin in the capped fritted column and incubated for 45 min (15 min included agitation by nutation), then the column was drained and washed with 2 x 1 CV of 1x coupling buffer. Residual unconjugated iodoacetamide groups on the resin were capped by treatment with cysteine methylester (CysOMe). 500µL of HP1α-GG-NpuN-6xHis, HP1α(1-176)-GG-NpuN-6xHis or HP1α(I163E)-GG-NpuN-6xH at a concentration of 50 µM was added to 125µL of the NpuC resin in a small fritted column and incubated on a nutator for 5min. The flowthrough was collected and the resin washed with 4 CV of binding buffer (100 mM phosphate, 1 mM EDTA, 1 mM TCEP, pH 7.2) with 500 mM NaCl, 4 CV of binding buffer with 300 mM NaCl and 4CV of binding buffer with 150 mM NaCl. Then 1 CV of *in situ* EPL buffer (50 mM MPAA, 200 mM

MESNa, 100 mM phosphate, 150 mM NaCl, 10 mM TCEP, 1 mM EDTA, pH 7.7-7.9) containing 1 mM of peptide P1 or P2 was added to the resin and the column was capped and incubated on a nutator for 16-18 h. The eluate was collected and the column further washed with 3 x 1 CV of intein column elution buffer (200 mM MESNa, 100 mM phosphate, 150 mM NaCl, 10 mM TCEP, 1 mM EDTA, pH 7.2). All fractions were analyzed by SDS-PAGE (**Figures 2 and 3**). Elution fractions were pooled and purified by gel filtration using a Superdex S200 10/300GL column using HP1 gel filtration buffer (**Supplementary Figures 4c and 8d**). Fractions with the labeled/dimerized protein were analyzed by SDS-PAGE, pooled and concentrated to 10-20 μM. 20 % glycerol was added, the final concentration and labeling efficiency was determined by UV spectroscopy using the extinction coefficient for HP1α at 280nm of ε_{280nm} = 29450 $M^{-1}cm^{-1}$ and for Atto532 at 532 nm of ε_{532nm} = 115000 $M^{-1}cm^{-1}$. Further the fluorescence emission spectra were analyzed by fluorometry (**Supplementary Figures 4f and 8f**) Proteins were mixed with glycerol and flash-frozen in 5-10 μL aliquots in liquid N_2 and stored at -80° C until use. Finally, both proteins were further analyzed by RP-HPLC and ESI-MS (HP1α(Atto532): Calculated MW = 23200.0 Da, observed MW = 23204.0 Da, HP1α_{cdm}: Calculated MW = 43944.65 Da, observed MW = 43947 Da, HP1α(I163E)-GG-NpuN-6xH: Calculated MW = 23213 Da, observed MW = 23221 Da (**Supplementary Figures 4e,f** & **8e**).

Single molecule protocols

Cleaning, silanization, and PEGylation of coverslips and glass slides and assembly of channels

For the assembly of reaction chambers, published protocols were followed [5], with some changes. In short, coverslips (24 x 40 mm, thickness 1.5) and glass slides (76 x 26 mm, containing two rows of four drilled holes) were placed in glass staining jars. They were sonicated for 20 minutes in 10% alconox, rinsed with deionized miliQ water, sonicated in acetone and finally ethanol. Then, they were dried and placed in a "piranha etch" solution (concentrated sulfuric acid / hydrogen peroxide, ratio of 3:1) for 1 h. The slides and coverslips were rinsed with deionized miliQ water, sonicated for 10 min in acetone and subsequently

silanized with 3 % (3-Aminopropyl)triethoxysilane (APTES) in acetone for 5 min, while shaking. The reaction was quenched with deionized miliQ water and the coverslips and slides were dried with nitrogen gas and stored for later use. Four flow channels were made on the slide, each connecting two drilled holes, using double-sided adhesive tape (100 μm thickness) as spacers. The channels were closed by placing a cleaned coverslip on top of the slide and epoxy glue was applied as a seal at both edges. Finally, a 20 μL pipette tip was glued to one side (for inset of tubing as exit port) and a 200 μL pipette tip (serving as a solvent reservoir) was glued to the other side of the channels. Subsequently, mPEG-succinimidyl carbonate containg 1% biotin-mPEG--succinimidyl carbonate, dissolved in 0.1 M sodium bicarbonate, pH 8.5 was prepared and 30 μl was injected into each channel and incubated 2-3 hours for passivation and formation of chromatin immobilization anchors. The channels were then washed with a total of 500 μl water and used on the same day.

smTIRFM imaging

For (objective-type) smTIRFM imaging, a fully automated Nikon Ti-E inverted fluorescence microscope, controlled by NIS-elements, equipped with a CFI Apo TIRF 100x Oil immersion objective (NA 1.49) and equipped with a manually controlled TIRF illuminator arm was employed. Data was acquired using an iXon EMCCD camera (Andor), with one pixel corresponding to 160 nm. Excitation light was provided from a home-built laser bench. Employed lasers were: A coherent OBIS 640 LX (640 nm, 40 mW), coherent OBIS 488 LX (488 nm, 50 mW) and a TECGL Series WS 532 (532 nm, 30 mW). Wavelength selection and switching of the excitation light was performed using an acousto-optical tunable filter (AOTF) controlled by NIS-elements. For all measurements, light intensities of 20 W/cm^2 were employed for 532 nm and 40 mW/cm^2 for 640 nm. Under these conditions, single Atto532 fluorophores were found to bleach with a time constant of 30 s (**Supplementary Fig. 6a**), while Atto647N fluorophores remained stable for > 100 s. For imaging conditions (imaging buffer, see below) TIRF was reached at an angle around 63°. All measurements were

performed at an angle of 64°, resulting in a decay constant of the evanescent wave of 195 nm for 532 nm, as experimentally determined using published protocols (**Supplementary Fig. 6b**) [6].

For chromatin imaging experiments, the flow channel was washed with 50 µL T50 (10 mM Tris, 50 mM NaCl, pH 8.0), using an automated pump (WPI SP210IWZ syringe pump). The background level of fluorescence was recorded with both 532 nm and 640 nm excitation. 50 µL of 0.2 mg/mL neutravidin solution was then injected into the channel and incubated 5 min, followed by washing with 500 µL T50. Subsequently, biotinylated and Atto647N labeled chromatin arrays were flowed into the channel at a concentration of 500 pM, followed by 2 min incubation. The immobilization step was monitored by Atto647N emission, aiming for a coverage of 100-200 arrays in a 25 x 50 µm imaging area. Excess chromatin in solution as well as MMTV DNA and associated nucleosomes were removed by a washing step, and the buffer exchanged to imaging buffer (50 mM HEPES, pH 7.5, 130 mM KCl, 10% v/v glycerol, 0.005% v/v Tween 20, 2 mM Trolox, 3.2% (w/v) glucose, 1x glucose oxidase / catalase enzymatic oxygen depleting system). HP1α dilutions in imaging buffer were freshly prepared from a 100 nM stock and injected into the channel. Imaging of HP1α binding dynamics was performed at a framerate of 20 frames/s (50 ms integration time) using 532 nm excitation for 10'000 frames. Every 200 frames, the excitation was switched to 640 nm for one frame to record the positions of the chromatin arrays. These frames were used in data analysis to localize array positions and correct for stage drift. Once immobilized, chromatin arrays were used for measurements for maximal one hour, to avoid disintegration of the chromatin.

Image processing, single-molecule trace extraction and trace analysis

Movies of HP1α binding dynamics were processed with a custom-built Matlab (Mathworks) program suite. First, a background correction was applied by fitting a two dimensional Gaussian profile to the image (overwhelming background) and subtracted from each frame. Subsequently, chromatin array positions were determined by a thresholding / local maxima approach. Then, all images were aligned using the

chromatin array images (every 200th frame) to correct for stage drift. In a semiautomatic program, well isolated chromatin peaks were selected in the frames recorded with 640 nm excitation. The fluorescence intensity within a 2 pixel radius around the peak was integrated over the whole stack of images and extracted as a fluorescence intensity vs time trace, showing HP1α binding events. Every detected peak (532 nm excitation, corresponding to bound HP1α molecules) was automatically fitted to a two-dimensional Gaussian profile, and peak width as well as x,y positions were recorded. Peaks with a width exceeding the experimentally determined point spread function (PSF) for a single Atto532 dye molecule, as well as peaks whose maxima were offset from the determined chromatin position were excluded from further analysis. Traces were then filtered using a forward-backward non-linear filter to reduce noise, while preserving sharp transitions[7]. Steps (binding events) were detected using a thresholding algorithm in a semi-automatic procedure, and on- and off-times were determined. All the fitted traces were further checked manually. Cumulative histograms were generated from traces corresponding to individual chromatin arrays (**Figure 4**) or from cumulative histograms from 100 traces (**Figures 2, 3 and 5**) and fitted to either single- or double-exponential kinetics.

Brownian dynamics model of chromatin binding kinetics

To understand the mechanism of the acceleration chromatin association with multivalent binders, we implemented a coarse-grained Brownian dynamics simulation[8] of binder – chromatin interactions (**Supplementary Fig. 10**). In this minimal model, a chromatin chain is represented by a spatially fixed string of 11 nm diameter spheres in a random conformation. Binders (either monomeric or dimeric) are represented by spheres with a diameter of 4 nm and can undergo Brownian motion, as described in the discretized Langevin equation:

$$\frac{\partial \mathbf{r}_i}{\partial t} = -\sum_{j}^{N} \frac{D_{ij}}{k_B T} \cdot \nabla_j U_{ij} + \xi_i \qquad (1)$$

where the sum is performed over all molecules N, r_i are the positions of the molecule under consideration, D_{ij} is the diffusion coefficient $D_{ij} = \delta_{ij} k_B T / (6\pi\eta a)$, with k_B the Boltzmann constant, T the temperature, η the solvent viscosity, a the radius of the diffusing particle and δ_{ij} the Kronecker delta. ξ_l is a stochastic term with the following properties:

$$\langle \xi \rangle = 0 \qquad \langle \xi_i \xi_j \rangle = 2D_{ij}\delta t \tag{2}$$

with δt the time step of the simulation. Finally, U_{ij} is a potential function describing all intermolecular interactions:

$$U_{ij} = k_B T \varepsilon \sum_{\alpha\beta ij} \omega_{\alpha\beta ij}^{LJ} \left[\left(\frac{a_\alpha + a_\beta}{r_{ij}} \right)^{12} - 2 \left(\frac{a_\alpha + a_\beta}{r_{ij}} \right)^{6} \right] + \frac{k_B T}{2a^2} \kappa \sum_{\alpha\beta ij} \omega_{\alpha\beta ij}^{C} \left(r_{ij} - (a_\alpha + a_\beta) \right)^2 \tag{3}$$

where the first term is a 6-12 Langevin potential, describing steric repulsion and nonspecific interactions between binders and chromatin, and the second term is a harmonic force, modeling noncovalent interactions. ε and κ indicate the strength of the respective potentials. Here the coefficients α and β denote the two different types of molecules, nucleosomes and binders respectively. The coefficients $\omega_{\alpha\beta ij}^{LJ}$ and $\omega_{\alpha\beta ij}^{C}$ are matrix elements (1 or 0) describing which molecules interact with each other. Lennard-Jones interactions only occur between binders and nucleosomes, whereas harmonic forces only occur between bound molecules (e.g. binder – binder interactions in dimers, or binders-nucleosomes if specifically bound). If binding (or dissociation) occurs, the respective matrix elements $\omega_{\alpha\beta ij}^{C}$ are updated to reflect the new situation. The Langevin equation is evaluated in time steps $\delta t = 0.002\ \tau_D$, with τ_D being the binder diffusion time $\tau_D = 1/D$. Binding is evaluated in a second time interval $\tau_0 = 0.05\ \tau_D$, in a Monte Carlo process. Each time interval τ_0, random number Ξ between 0 and 1 are determined and the connectivity matrix $\omega_{\alpha\beta ij}^{C}$ is re-evaluated by the following rules:

$$\omega^{C}_{\alpha\beta ij}(\mathrm{t})=\begin{cases}\begin{cases}1\, if\ \Xi<e^{-\Delta E_B/k_BT}\\0\, if\ \Xi>e^{-\Delta E_B/k_BT}\end{cases} & if\quad \omega^{C}_{\alpha\beta ij}(\mathrm{t}-\tau_0)=0\quad \text{and}\quad r_{ij}<r_{RXN}\\ \begin{cases}1\, if\ \Xi<e^{-\Delta E_{UB}/k_BT}\\0\, if\ \Xi>e^{-\Delta E_{UB}/k_BT}\end{cases} & if\quad \omega^{C}_{\alpha\beta ij}(\mathrm{t}-\tau_0)=1\end{cases} \tag{4}$$

with r_{RXN} being the interaction radius of nucleosomes, ΔE_{UB} an energy barrier for unbinding and ΔE_B the energy barrier for binding (**Supplementary Fig. 10a**). In addition, each nucleosome can only interact with one binder at a time.

For the simulations, 200 binders were placed at random positions into a box of 200 nm x 200 nm x 200 nm with periodic boundary conditions and either dimerized using harmonic forces between pairs of binders or left as monomers. Binding parameters were systematically varied. For displayed simulations, the following parameters were used: r_{RXN} = 11 nm, E_B = 7.5, E_{UB} = 8.5, ε = 1, κ = 200 (**Supplemental figures 10a, b**). For evaluation of binding kinetics, the system was let to relax for 50'000 steps. Then, 100 first-passage times to the first binding event were determined for monomers and dimers, histograms were generated and evaluated. For 100 dimeric binders, the thus determined association kinetics were found to be around twice as fast, as for 200 monomers (**Figure 4e**). Dimers diffuse more slowly (with a smaller diffusion coefficient), and due to their lower concentration the collision frequency with chromatin is much reduced. In contrast however, the probability of binding is much increased during a transient collision, and non-specific interactions (Lennard-Jones potential) result in longer contact times, thereby further increasing the chance of binding compared to monomers. Dissociation kinetics were also determined. In such an experiment, a monomeric or dimeric binder was placed on a random nucleosome and the system was simulated until the binder dissociated and diffused a minimum of 50 nm from the closest nucleosome. Here, for monomeric binders, multiple dissociation and re-binding events result in monoexponential kinetics (**Supplementary Fig. 10c**) which scale with the binding energies E_B and E_{UB}. For dimeric binders, double-exponential kinetics are observed (**Supplementary Fig. 10c**) if the following conditions are met: after the initial (monovalent) binding event, direct dissociation must occur with higher probability than

engagement of a nucleosome with the second binding domain (bivalent binding). This can either be achieved by reducing E_B relative to E_{UB} or to introduce further geometric constraints for multivalent interactions (e.g. reducing r_{RXN}). As shown in Supplementary Figure 10, with the above mentioned parameters, double exponential kinetics are observed for dimeric binders, with the slow phase arising from bivalent binding. Such behavior is also found for HP1α molecules in our single molecule studies.

Kinetic modeling

Stochastic modeling of HP1 binding kinetics was performed based on the kinetic scheme shown in **Figure 4f** using a Matlab (Mathworks) implementation of the Gillespie Stochastic Simulation Algorithm [9]. The differential equations of the kinetic system are given below:

$$\frac{d[\mathrm{HP1}]}{dt} = -k_{\mathrm{dim}}[\mathrm{HP1}]^2 + \mathrm{k}_{\mathrm{mono}}[\mathrm{HP1}_2] - k_{assoc}[\mathrm{HP1}][\mathrm{DNA}] + k_{dissoc}[\mathrm{HP1...DNA}] \quad (5)$$

$$\frac{d[HP1...DNA]}{dt} = -k_{dissoc}[\mathrm{HP1...DNA}] + k_{assoc}[\mathrm{HP1}] - k_{bind}[\mathrm{HP1...DNA}][\mathrm{H3K9me3}] + k_{release}[\mathrm{HP1\text{-}H3K9me3}] - k_{\mathrm{dim}}[\mathrm{HP1...DNA}]^2 + k_{mono}[\mathrm{HP1}_2\mathrm{...DNA}] \quad (6)$$

$$\frac{\mathrm{d}[\mathrm{HP1\text{-}H3K9me3}]}{dt} = -k_{release}[\mathrm{HP1\text{-}H3K9me3}] + k_{bind}[\mathrm{HP1...DNA}][\mathrm{H3K9me3}] - k_{\mathrm{dim}}[\mathrm{HP1\text{-}H3K9me3}][\mathrm{HP1...DNA}] + k_{mono}[\mathrm{HP1}_2\mathrm{...DNA}] \quad (7)$$

$$\frac{d[\mathrm{HP1}_2]}{dt} = -k'_{assoc}[\mathrm{HP1}_2] + k_{dissoc}/\alpha[\mathrm{HP1}_2\mathrm{...DNA}] - k_{mono}[\mathrm{HP1}_2] + k_{\mathrm{dim}}[\mathrm{HP1}]^2 \quad (8)$$

$$\frac{d[\mathrm{HP1}_2\mathrm{...DNA}]}{dt} = -k_{dissoc}/\alpha\,[\mathrm{HP1}_2\mathrm{...DNA}] + k'_{assoc}[\mathrm{HP1}_2] - 2k_{bind}[\mathrm{HP1}_2\mathrm{...DNA}][\mathrm{H3K9me3}] + k_{release}[\mathrm{HP1\text{-}H3K9me3}] - k_{mono}[\mathrm{HP1}_2\mathrm{...DNA}] + k_{\mathrm{dim}}[\mathrm{HP1...DNA}]^2 \quad (9)$$

$$\frac{d[\mathrm{HP1_2\text{-}H3K9me3}]}{dt} = -k_{release}[\mathrm{HP1_2\text{-}H3K9me3}] + 2k_{bind}[\mathrm{HP1_2...DNA}][\mathrm{H3K9me3}] - k_{mono}[\mathrm{HP1_2...DNA}] + k_{\mathrm{dim}}[\mathrm{HP1\text{-}H3K9me3}][\mathrm{HP1...DNA}] - k_{biv}[\mathrm{HP1_2\text{-}H3K9me3}][\mathrm{HP1...DNA}] + 2k_{release}[\mathrm{HP1_2\text{-}H3K9me3_2}] \quad (10)$$

$$\frac{d[\mathrm{HP1_2\text{-}H3K9me3_2}]}{dt} = -2k_{release}[\mathrm{HP1_2\text{-}H3K9me3_2}] + k_{biv}[\mathrm{HP1_2\text{-}H3K9me3}][\mathrm{HP1...DNA}] \quad (11)$$

Kinetics were simulated using 1 nM HP1α molecules and the kinetic parameters reported in **Supplementary table 1**. Throughout the simulation, the concentration of unbound HP1 molecules was kept constant to attribute for open system boundaries.

All the DNA and chromatin associated states were considered as “bound” and used to generate a trace resembling a single-molecule experiment. The trace was resampled to a 50 ms time resolution by averaging signals over this time period. Finally, the constructed traces were fed into our analysis software and rate constants were extracted. A semi-automated fitting procedure was employed to generate a parameter-set (**Supplementary table 1**) that describes the rapid component of the measured data well.

Supplementary references

1. Fierz, B. et al. Histone H2B ubiquitylation disrupts local and higher-order chromatin compaction. *Nat Chem Biol* **7**, 113-9 (2011).
2. Stavropoulos, G., Gatos, D., Magafa, V. & Barlos, K. Preparation of polymer-bound trityl-hydrazines and their application in the solid phase synthesis of partially protected peptide hydrazides. *Lett Pept Sci* (1996).
3. Nguyen, U.T. et al. Accelerated chromatin biochemistry using DNA-barcoded nucleosome libraries. *Nat Methods* **11**, 834-40 (2014).
4. Vila-Perello, M. et al. Streamlined expressed protein ligation using split inteins. *J Am Chem Soc* **135**, 286-92 (2013).
5. Roy, R., Hohng, S. & Ha, T. A practical guide to single-molecule FRET. *Nat Methods* **5**, 507-16 (2008).
6. Fish, K.N. Total internal reflection fluorescence (TIRF) microscopy. *Curr Protoc Cytom* **Chapter 12**, Unit12 18 (2009).
7. Chung, S.H. & Kennedy, R.A. Forward-backward non-linear filtering technique for extracting small biological signals from noise. *J Neurosci Methods* **40**, 71-86 (1991).
8. Sing, C.E., Olvera de la Cruz, M. & Marko, J.F. Multiple-binding-site mechanism explains concentration-dependent unbinding rates of DNA-binding proteins. *Nucleic Acids Res* **42**, 3783-3791 (2014).
9. Gillespie, D.T. Exact Stochastic Simulation of Coupled Chemical-Reactions. *J Phys Chem* **81**, 2340-2361 (1977).
